# Novel hybrid kepler optimization algorithm for parameter estimation of photovoltaic modules

**DOI:** 10.1038/s41598-024-52416-6

**Published:** 2024-02-11

**Authors:** Reda Mohamed, Mohamed Abdel-Basset, Karam M. Sallam, Ibrahim M. Hezam, Ahmad M. Alshamrani, Ibrahim A. Hameed

**Affiliations:** 1 Zagazig University, Zagazig, 44519 Sharqiyah Egypt; 2 Department of Computer Science, University of Sharjah, Sharjah, United Arab Emirates; 3 School of IT and Systems, Faculty of Science and Technology, University of Canberra, Canberra, Australia; 4 Statistics & Operations Research Department, College of Sciences, King Saud University, 11451 Riyadh, Saudi Arabia; 5 Department of ICT and Natural Sciences, Norwegian University of Science and Technology (NTNU), Ålesund, Norway

**Keywords:** Engineering, Mathematics and computing

## Abstract

The parameter identification problem of photovoltaic (PV) models is classified as a complex nonlinear optimization problem that cannot be accurately solved by traditional techniques. Therefore, metaheuristic algorithms have been recently used to solve this problem due to their potential to approximate the optimal solution for several complicated optimization problems. Despite that, the existing metaheuristic algorithms still suffer from sluggish convergence rates and stagnation in local optima when applied to tackle this problem. Therefore, this study presents a new parameter estimation technique, namely HKOA, based on integrating the recently published Kepler optimization algorithm (KOA) with the ranking-based update and exploitation improvement mechanisms to accurately estimate the unknown parameters of the third-, single-, and double-diode models. The former mechanism aims at promoting the KOA’s exploration operator to diminish getting stuck in local optima, while the latter mechanism is used to strengthen its exploitation operator to faster converge to the approximate solution. Both KOA and HKOA are validated using the RTC France solar cell and five PV modules, including Photowatt-PWP201, Ultra 85-P, Ultra 85-P, STP6-120/36, and STM6-40/36, to show their efficiency and stability. In addition, they are extensively compared to several optimization techniques to show their effectiveness. According to the experimental findings, HKOA is a strong alternative method for estimating the unknown parameters of PV models because it can yield substantially different and superior findings for the third-, single-, and double-diode models.

## Introduction

The demand for energy in various countries is continuously on the rise as a consequence of factors such as their expanding industries and rapidly expanding populations^1,2^. Additionally, fuel depletion and environmental pollution are the main drawbacks of traditional fossil fuel resources, which have recently prompted scientists to find a new energy source that can save energy without having any negative effects on the environment^3^. Therefore, the scientists thought of alternative renewable energy sources, like wind, geothermal, solar, and hydroelectricity, for generating abundant energy without increasing environmental pollution. Among renewable energy sources, solar photovoltaic (PV) has recently won significant interest over the previous decades due to several merits like low computational cost, low operational expenses, high density of power, and low maintenance^3–5^. The PV panel consists of PV cells related in parallel and series. The manufacturing process and environmental conditions like temperature and light intensity influence the panel output^3^.

There is a significant information gap in the PV module parameters provided by vendors and manufacturers, which stands as an obstacle to accurately simulating the PV modules^3^. An ideal electrical equivalent circuit for a solar PV cell would include a diode, a current source, and some resistors^3^. PV cell models are split into three kinds, depending on whether they include two diodes, single diodes, or triple diodes to accurately estimate the cell's I–V curve^3^. SDM is considered the simplest model because it includes only five unknown parameters; on the contrary, DDM includes seven known parameters and is considered more precise than SDM. TDM includes nine unidentified parameters and is considered the highest accurate model due to its ability to address the effects of grain boundaries and leakage current coefficients^3,6^. Over the last few decades, estimating those parameters has been classified as a complex non-linear optimization problem^7–11^. This problem has been extensively tackled in the literature by either traditional techniques or approximation techniques^9^. The traditional techniques could not accurately solve this problem due to several reasons, like differentiability and convexity, relying substantially on the initial solution, and falling easily in local optima^12^. Therefore, the metaheuristic algorithms also referred to as approximation techniques, have been adapted to accurately solve this problem for accurately modeling solar PV systems. This interest is due to their ability to accurately solve several complicated problems in a reasonable time^13–20^. For example, Abdel-Basset et al.^21^ designed a new metaheuristic optimizer, namely the nutcracker optimizer, for solving continuous optimization problems. This optimizer was validated using three CEC benchmarks and compared to several metaheuristic algorithms. The experimental findings showed its effectiveness. In the same study, this optimizer was applied to some engineering design problems and proved its effectiveness. In addition, this algorithm was applied to some optimization problems, such as the parameter identification of PV^22^ and Distribution of Fresh Agricultural Products^23^, and could achieve outstanding outcomes.

In^3^, the Northern Goshawk Optimization (NGO) algorithm was adapted for extracting the TDM’s unidentified parameters. NGO’s performance was investigated using three different PV modules and compared to several competing optimizers to show its efficacy. According to the experimental outcomes, NGO could perform better than all the others for convergence speed and final accuracy. The tree seed algorithm (TSA) was also applied to tackle this problem under the RMSE as an objective function^1^. This algorithm was validated using the STM module and compared to some of the recently-published metaheuristics. The comparison shows its effectiveness over the others. In addition, the seagull optimization algorithm (SOA) was hybridized with the memory saving to preserve the historical best solution, the cosine function to equilibrium between exploration and exploitation operators, and the differential mutation strategy to escape out of local minima^24^. This hybrid variant of SOA was known as HSOA and employed to identify the unknown values of various parameters in SDM and DDM for various PV modules.

The war strategy optimization (WSO) algorithm has been recently applied to solve this problem with the aid of the NR method to enhance the quality of the obtained solutions^25^. According to the experimental findings, WSO performed better than several metaheuristic algorithms. The Hunger Game search algorithm was combined with the Laplacian strategy and Nelder-Mead simplex to present a new variant, abbreviated LNMHGS, which could achieve equilibrium between exploration and exploitation operators when applied to identify the unknown values of various parameters in three PV models^26^. In^27^, the exploitation operator of the teaching–learning-based optimizer was integrated with the exploration operator of the artificial bee colony to design a new optimizer, denoted as TLABC. This optimizer was applied to find the unidentified parameters of both DDM and SDM. Also, it was compared to several rival algorithms to reveal its effectiveness. The heap-based optimizer was also developed to search for the unidentified parameters of three PV models^28^. In^29^, chaotic maps were utilized as an alternative to the random number that was generated in order to exchange between generating a random individual within the search space and updating the current position in accordance with the updating core of the SMA. The authors state that the chaotic maps provide SMA with a more effective exploratory pattern. In addition, the Nelder-Mead simplex approach was incorporated into SMA in order to enhance its convergence speed and achieve better results with fewer function evaluations. CCNMSMA was the abbreviation of this version of SMA that was employed to find the unknown parameters of PV models. The generalized normal distribution optimizer was enhanced by utilizing two powerful methods, which led to the development of a new optimizer known as IGNDO^30^. This optimizer was utilized in the process of estimating the unknown parameters of TDM. In comparison to the results obtained by many other algorithms, it was able to attain remarkable results. However, this technique necessitates a somewhat higher level of computing expense. In addition to this, it still has a problem with sluggish convergence speed because it takes several function evaluations before it can converge to the results that are required. Rawat et al.^31^ adapted the grey wolf optimizer to identify those unknown parameters. Table 1 reviews some recently proposed metaheuristic algorithms for estimating the unknown parameters of SDM, DDM, and TDM.

**Table 1:** Some metaheuristic algorithms proposed recently for the parameter estimation of PV models.

Year	Algorithm	OF	Modelling	Contributions	References
2023	Improved moth flame algorithm	RMSE	SDM; DDM	This study improved the moth flame algorithm using local escape operators to enhance its exploration operator and population diversity. This algorithm could achieve outstanding outcomes in comparison to some algorithms, but it required a huge number of function evaluations, up to 125,000	^32^
2023	Hybrid grey wolf optimization	RMSE	DDM	This study combined the analytical technique and grey wolf algorithm to present a strong variant for tackling this problem	^33^
2023	Chaos game optimization algorithm	RMSE	SDM; DDM; TDM	In this study, the Chaos game optimization is combined with the least squares (LS) estimator to aid in accelerating the convergence speed and achieving better outcomes	^34^
2023	Squirrel search algorithm	RMSE	SDM; DDM	This study adapted the squirrel search algorithm for estimating the unknown parameters of SDM and DDM by minimizing the root mean squared error between the measured and estimated data	^35^
2023	Growth Optimizer	RMSE	SDM; DDM	The growth optimizer was developed to find the unknown parameters for two different PV modules, namely KKC and RTC. This algorithm consumed a huge number of function evaluations for solving this problem	^36^
2023	L-SHADE	RMSE	SDM	In this study, a parameter decomposition technique was used to alleviate this problem's complexity. Then, the L-SHADE was applied to estimate the unknown values for those parameters. This presented technique was called the L-SHADE with parameter decomposition (L-SHADED). This technique was assessed using two different PV modules and compared to some competitors to reveal its effectiveness	^37^
2023	Opposition-Based Initialization Particle Swarm Optimization	RMSE	SDM	In this study, the particle swarm optimization was improved using the opposition-based theory for better solving the parameter identification problem of SDM	^38^
2023	Chimp optimization algorithm	RMSE	SDM, DDM, TDM	The Chimp optimization algorithm was employed in this study to minimize the RMSE between the measured and estimated data for finding the optimal values of the unknown parameters in three different PV models	^39^
2023	Harris Hawks optimizer	RMSE	SDM, DDM, TDM	the Harris Hawks optimization algorithm was enhanced by the fractal maps to propose a new technique, namely FCHHHO. This technique was used to identify the unknown parameters of the RTC France solar cell and PWP PV module based on SDM, DDM, and TDM	^4^
2023	Artificial humming bird optimizer	RMSE, NR, Lambert W function	SDM, DDM	This algorithm was adapted for tackling the parameter identification of PV models under three different OFs	^40^
2023	Improved Particle Swarm Optimization	RMSE	SDM, DDM	Qaraad et al. improved the particle swarm optimization using a local search method to avoid stagnation into local minima and quadratic interpolation to improve the convergence speed. This improved variant was referred to as QPSOL and applied to determining the unidentified parameters of SDM and DDM	^41^
2023	Ranking-based artificial gorilla troop optimizer (RGTO)	RMSE	TDM	In this study, the artificial gorilla troop optimizer (GTO) was improved using two mechanisms to strengthen its search ability to prevent stagnation into local minima and accelerate the convergence rate. This improved variant was called RGTO and applied to solve the parameter estimation of TDM and fuel cells	^42^
2023	Amended reptile search algorithm	RMSE	SDM, DDM	In this study, the reptile search algorithm (RSA) was enhanced by the opposition-based theory to avoid falling into local minima and accelerate the convergence speed. In addition, this improved RSA was incorporated with the Cauchy mutation strategy to further improve the exploration operator. This improved variant was called OBL-RSACM and applied to estimate the unknown parameters of SDM, DDM, and TDM	^43^
2023	Eight metaheuristic algorithms	RMSE	SDM	Sharma et al. studied the performance of eight metaheuristic algorithms for solving the parameter identification problem of four PV modules and cells. The experimental findings show that the coot-bird optimization technique is the best for the RTC France solar cell and the LSM20 PV module, while the wild horse optimizer could be the best for the SS2018 and Solarex MSX-60 PV modules	^44^
2023	Improved cheetah optimizer (ICO)	RMSE	SDM, DDM	Memon proposed a new optimization technique, namely ICO, based on improving the cheetah optimizer for accurately solving this problem	^45^

The existing metaheuristic optimization techniques for the parameter estimation problem of PV models suffer from at least one of the following drawbacks: Falling into local optima, slow convergence speed, and high computational cost. To alleviate these shortcomings, a new optimization technique based on improving the Kepler optimization algorithm (KOA) using two effective mechanisms, namely the ranking-based update and exploitation improvement mechanisms, for accurately estimating the unknown parameters of the third, single, and double-diode models is presented in this study. The KOA's exploration operator is encouraged by the first mechanism to reduce the likelihood of getting stuck in local optima, and its exploitation operator is strengthened by the second mechanism to accelerate the convergence to the approximation solution.

Recently, the Kepler optimization algorithm (KOA) was introduced to handle continuous optimization problems^46^. This algorithm was influenced by Kepler's laws on the motion of planets. These laws demonstrate that four variables can influence the path of the planet around the sun. These elements are reflected in a planet's gravitational force, position, mass, and orbital velocity. KOA views each planet as a candidate solution, and during a planet's motion, it may estimate a new solution to the optimization problem. Those planets closest to the sun improve the exploitation operator, while the others attempt to explore the search space. To the best of our knowledge, this algorithm has not yet been applied to finding the unidentified parameters of DDM, SDM, and TDM. Therefore, in this paper, KOA is applied to this problem to reveal its effectiveness. In addition, it is combined with two effective mechanisms to design a new strong variant called HKOA with better performance to extract the near-optimal values for those parameters more accurately. Both KOA and HKOA are validated using six PV modules and contrasted with several state-of-the-art optimizers to reveal their effectiveness. HKOA, according to the experimental findings, is a powerful alternative since it is able to achieve outstanding outcomes for the utilized PV cells and modules in comparison to all rival optimizers. The key contributions to this study are listed below:

- Developing the newly proposed KOA for finding the unidentified parameters of DDM, TDM, and SDM.
- Hybridizing KOA with two effective mechanisms to design a new better variant, namely HKOA, with a better ability to accelerate convergence speed and avoid local optima for estimating those unknown parameters with greater precision.
- Assessing both KOA and HKOA using the RTC France solar cell and five PV modules
- Comparing both KOA and HKOA to several competitors to reveal their effectiveness.
- Based on the experimental findings, HKOA is better than all the compared techniques since it could produce significantly different and superior outcomes.

The following sections are structured as: Section "Problem formulation" discusses the PV models’ mathematical model, Section "Kepler optimization algorithm (KOA)" overviews the classical Kepler optimization algorithm, Section "Proposed algorithm" explains the proposed algorithm, Section "Results and discussion" reports and discusses the experimental findings, and Section "Conclusions and Future Works" states the Conclusion and Future work.

## Problem formulation

This paper is presented to identify the unknown parameters of three photovoltaic models, including TDM, DDM, and SDM. The mathematical models of those PV models are presented in the rest of this section.

### SDM

**Figure 1:**
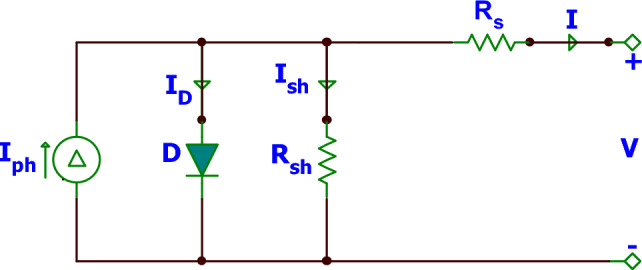
The equivalent circuit diagram for SDM.

Figure 1 displays the SDM’s electrical circuit. The output current of SDM can be computed by Kirchhoff’s Current Law (KCL), as defined in the following formula^25,27^:

1 $$I={I}_{ph}-{I}_{D}-{I}_{sh},$$

where $I$ represents the solar cell’s current, and ${I}_{ph}$ represents the photocurrent^47^. Diode current, indicated by the symbol ${I}_{D}$, can be computed using the following formula^25,27^:

2 $${I}_{D}={I}_{sd}\left({exp}\left(\frac{V+I*{R}_{s}}{n*{V}_{t}}\right)-1\right),$$

where ${I}_{sd}$ represents the reverse saturation current of the diode, $V$ indicates the output voltage, *R*_*s*_ refers to the series resistance, and *n* is the ideality factor. ${V}_{t}$ is estimated by:

3 $${V}_{t}=\frac{k*T}{q},$$

where $T$ is a symbol to refer to the temperature (Kelvin), $k$ is a symbol used to refer to the Boltzmann constant, and $q$ is a symbol used to stand for the charge of the electron, ${I}_{sh}$ presented in Eq. (1) is defined by:

4 $${I}_{sh}=\frac{V+I*{R}_{s}}{{R}_{sh}},$$

where ${R}_{sh}$ represents the shunt resistance. The following is a general formula that can be used to calculate $I$:

5 $$I={I}_{ph}-{I}_{sd}\left({exp}\left(\frac{q*\left(V+I*{R}_{s}\right)}{n*k*T}\right)-1\right)-\frac{V+I*{R}_{s}}{{R}_{sh}}.$$

In order to accurately find the I-V characteristic of the SDM, It is necessary to determine the unknown values of the following parameters: ${I}_{ph}, {I}_{sd}, n, {R}_{s}, {and R}_{sh}$. This estimation process could be represented as an optimization problem, and hence the optimization methods could be utilized to solve it.

### DDM

**Figure 2:**
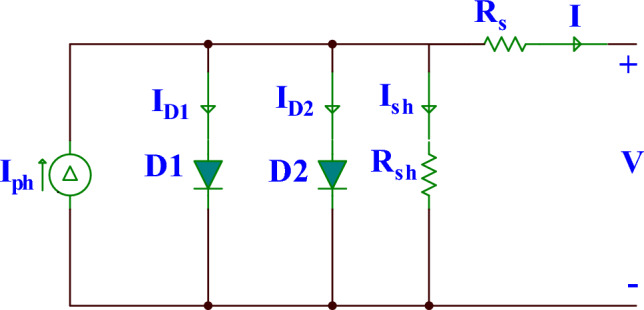
The equivalent circuit diagram for DDM.

DDM was developed to provide a robust substitute for SDM in conditions where the SDM is suboptimal, such as in low irradiance environments^48^. The DDM is depicted in Fig. 2 and is made up of two diodes. The first diode serves as a rectifier, while the second compensates for recombination current and solar cell imperfections. The mathematical formula that could be used to compute the DDM’s output current is as follows^25,27^:

6 $$I={I}_{ph}-{I}_{sd1}\left({exp}\left(\frac{V+I*{R}_{s}}{{n}_{1}*{V}_{t}}\right)-1\right)-{I}_{sd2}\left({exp}\left(\frac{V+I*{R}_{s}}{{n}_{2}*{V}_{t}}\right)-1\right)-\frac{V+I*{R}_{s}}{{R}_{sh}},$$

where ${I}_{sd1}$ is the current through the first diode and ${I}_{sd2}$ is the current through the second diode. ${n}_{1}$ and ${n}_{2}$ are the ideality factors. There are seven unknown parameters in this formula: ${I}_{ph}$*, *${I}_{sd1}$*, *${I}_{sd2}$*, *${R}_{s}$*, *${R}_{sh}$*, *${n}_{1}$*, and *${n}_{2}$*,* which need to be accurately estimated to reliably find the I-V characteristic of the DDM.

### TDM

**Figure 3:**
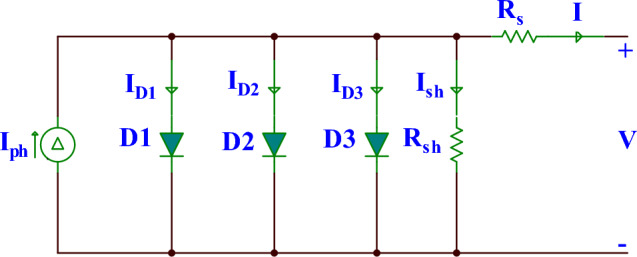
The equivalent circuit diagram for TDM.

As illustrated in Fig. 3, a photocurrent source, ${I}_{ph}$, a shunt resistor ${R}_{sh}$, three parallel diodes, and a series resistance ${R}_{s}$ make up the TDM. The TDM’s output current could be estimated by:

7 $$I={I}_{ph}- {I}_{sd1}\left(exp\left(\frac{V+I{*R}_{s}}{{V}_{t}*{n}_{1}}\right)-1\right)- {I}_{sd2}\left(exp\left(\frac{V+I{*R}_{s}}{{V}_{t}*{n}_{2}}\right)-1\right)- {I}_{sd3}\left(exp\left(\frac{V+I{*R}_{s}}{{V}_{t}*{n}_{3}}\right)-1\right)-\frac{V+I*{R}_{s}}{{R}_{sh}},$$

where ${I}_{sd3}$ is the current through the third diode, and ${a}_{3}$ represents the third diode’s ideality factor. The TDM’s mathematical model has seven unknown parameters that need to be accurately estimated to maximize the performance of the solar cell. These parameters are namely ${I}_{ph}, {I}_{sd1},{I}_{sd2},{I}_{sd3}, {R}_{s},{R}_{sh},{a}_{1}, {a}_{2}, {\mathrm{ and }a}_{3}$. From above, it is clear that various PV models have different numbers of parameters, where SDM has five parameters, DDM has seven, and TDM has nine. The characteristics of each model are different, and hence the optimization algorithm that could find the near-optimal parameter for a model is not necessarily able to have the same performance for the other models. Therefore, in this study, we strive to design an alternative optimization technique with strong exploration and exploitation operators for estimating those unknown parameters for three PV models.

## Kepler optimization algorithm (KOA)

**Figure 4:**
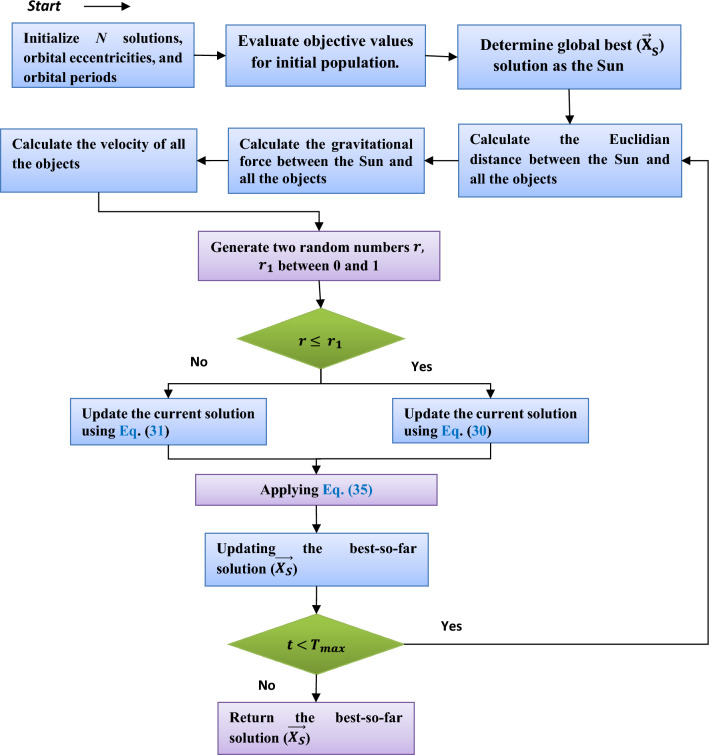
KOA’s Flowchart.

Recently, a new metaheuristic-based technique, namely the Kepler optimization algorithm (KOA), was presented to tackle continuous optimization problems. This algorithm was inspired by Kepler’s laws of planetary motion. These laws have shown that four factors could control the path of the planet around the sun. These factors are represented in the gravitational force, position, mass, and orbital velocity of a planet. KOA considers each planet as a candidate solution, and during the motion of a planet, it could estimate a new solution for the optimization problem. Those planets near the sun maximize the exploitation capabilities, while the others enhance the exploration capabilities. In brief, analogous to the other metaheuristic algorithms, KOA is based on two operators: exploitation and exploration. The KOA’s flowchart is depicted in Fig. 4. The KOA’s mathematical model is discussed in detail next.

**Step 1**: **Initialization step**

The KOA, in the beginning, distributes $N$ planets within the search space, where each planet is comprised of $d$ dimensions. The formula that is used to distribute the planets is mathematically formulated as follows:

8 $${X}_{i}^{j}={X}_{i,low}^{j}+r\times \left({X}_{i,up}^{j}-{X}_{i,low}^{j}\right), \left\{\begin{array}{c}i=\mathrm{1,2},\dots ,N\\ j=\mathrm{1,2},\dots ,d\end{array},\right.$$

where ${X}_{i}$ refers to the *ith* planet; ${X}_{i,low}^{j}$ and ${X}_{i,up}^{j}$ are the search limits of the *j*th dimension, respectively. $r$ is a number selected randomly in the range (0, 1). In addition, KOA has some parameters, like the orbital eccentricity ($e$) and orbital period (T), that need to be initialized before starting the optimization process. The orbital eccentricity ($e$) could be initialized by:

9 $${e}_{i}=r, i=1,\dots ,N$$

Finally, *T* for each $it$ h planet could be initialized by:

10 $${T}_{i}=\left|rn\right|, i=1,\dots ,N,$$

where $rn$ is a number selected randomly according to the normal distribution.

**Step 2: Defining** $$\mathbf{F}$$

The planets' motions around the Sun are governed by gravity, which is considered the fundamental force in the universe. The gravity of each planet varies in accordance with its mass. It's important to note that a planet's speed is affected by the Sun's gravity. When a planet is closer to the Sun, its orbital speed increases, and when it is farther away, the speed decreases. The universal law of gravity, which specifies the strength of the pull between the Sun ${\overrightarrow{X}}_{S}$ and any planet ${\overrightarrow{X}}_{i}$, could be defined as follows:

11 $${{{\varvec{F}}}_{{\varvec{g}}}}_{i}\left(t\right)={e}_{i}\times \mu \left(t\right)\times \frac{{\overline{M} }_{s}\times {\overline{m} }_{i}}{{{\overline{R} }^{2}}_{i}+\varepsilon }+{r}_{1},$$

where $\varepsilon$ contains a small value to avoid division by zero, ${r}_{1}$ is a variable including values generated randomly between 0 and 1. ${\overline{M} }_{s}\mathrm{ and} {\overline{m} }_{i}$ denote the normalized values of the mass of both ${X}_{S} {\text{and}} {X}_{i}$, respectively; the mass ${M}_{s}$ and ${m}_{i}$ of both ${X}_{S} {\text{and}} {X}_{i}$ are respectively computed according to Eqs. (14) and (15); $\upmu$ is a fixed value to represent the universal gravitational constant;${e}_{i}$ indicates the eccentricity of a planet’s orbit; and ${\overline{R} }_{i}$ represents the normalized value of the Euclidian distance between ${X}_{i}$ and ${X}_{S}$. The Euclidian distance between ${X}_{i}$ and ${X}_{S}$ could be computed by:

12 $${R}_{i}\left(t\right)={\Vert {X}_{S}\left(t\right)-{X}_{i}\left(t\right)\Vert }_{2}=\sqrt{\sum_{j=1}^{d}{\left({\overrightarrow{X}}_{Sj}\left(t\right)-{\overrightarrow{X}}_{ij}\left(t\right)\right)}^{2}},$$

13 $${\overline{R} }_{i} =\frac{{R}_{i}(t)-{\text{min}}(R(t))}{{\text{max}}\left({\text{R}}({\text{t}})\right)-{\text{min}}(R(t))},$$

14 $${M}_{s}={r}_{2}\frac{{fit}_{s}\left(t\right)-worst(t)}{\sum_{k=1}^{N}\left({fit}_{k}\left(t\right)-worst\left(t\right)\right)},$$

15 $${m}_{i}=\frac{{fit}_{i}\left(t\right)-worst(t)}{\sum_{k=1}^{N}\left({fit}_{k}\left(t\right)-worst\left(t\right)\right)},$$

16 $${\mathrm{where}}\;fit_{s} (t) = best(t) = \mathop {\min }\limits_{{k \in \{ 1,2, \ldots ,N\} }} fit_{k} (t) ,$$

17 $$worst(t) = \mathop {\max }\limits_{{k \in \{ 1,2, \ldots ,N\} }} fit_{k} (t) ,$$

where ${r}_{2}$ is a number chosen at random in the range (0, 1). ${M}_{s}$ represents the mass of ${X}_{S}$, and ${m}_{i}$ represents the mass of ${X}_{i}$. $\mu (t)$ is defined by:

18 $$\mu \left(t\right)={\mu }_{0}\times {\text{exp}}\left(-\gamma \frac{t}{{T}_{max}}\right),$$

where $\gamma$ is a fixed value; ${\mu }_{0}$ includes an initial value; and $t$ and ${T}_{max}$ represent the current function evaluation and the maximum function evaluations, respectively.

**Step 3: Calculating an object’s velocity**

A planet's speed is calculated by its distance from the Sun. In other words, a planet's velocity rises as it draws nearer to the Sun and diminishes as it moves farther away. When a planet or other object approaches the Sun, the Sun's gravity is much stronger, so the planet attempts to accelerate in order to avoid being pulled in. In^46^, this behavior is simulated using the following mathematical model:

19 $${V}_{i}\left(t\right)=\left\{\begin{array}{ll} \ell \times \left({2{r}_{4}\overrightarrow{X}}_{i}-{\overrightarrow{X}}_{b}\right)+\ddot{\ell}\times \left({\overrightarrow{X}}_{a}-{\overrightarrow{X}}_{b}\right)+\left(1-{R}_{i-norm}\left(t\right)\right)\times \mathcal{F}\times {\overrightarrow{U}}_{1}\times \overrightarrow{{r}_{5}}\times \left({\overrightarrow{X}}_{i, up}-{\overrightarrow{X}}_{i, low}\right), &\quad if \, {R}_{i-norm}(t)\le 0.5\\ {r}_{4}\times L\times \left({\overrightarrow{X}}_{a}-{\overrightarrow{X}}_{i}\right)+\left(1-{R}_{i-norm}\left(t\right)\right)\times \mathcal{F} \times {U}_{2}\times \overrightarrow{{r}_{5}}\times \left({r}_{3}{\overrightarrow{X}}_{i, up}-{\overrightarrow{X}}_{i, low}\right), &\quad Else\end{array}\right.$$

20 $$\ell =\overrightarrow{U}\times \mathcal{M}\times \mathcal{L},$$

21 $${\mathcal{L}=\left[\mu \left(t\right)\times \left({M}_{S}+{m}_{i}\right)\left(\frac{2}{{R}_{i}(t)+\varepsilon }-\frac{1}{{a}_{i(t)}+\varepsilon }\right)\right]}^\frac{1}{2},$$

22 $$\mathcal{M}=\left({r}_{3}\times \left(1-{r}_{4}\right)+{r}_{4}\right),$$

23 $$\overrightarrow{U}=\left\{ {\begin{array}{*{20}l} 0 \hfill & {\overrightarrow {{r_{5} }} \le \overrightarrow {{r_{6} }} } \hfill \\ 1 \hfill & {Else} \hfill \\ \end{array} } \right.,$$

24 $${\mathcal{F}} = \left\{ {\begin{array}{*{20}l} 1 \hfill & {if \, r_{4} \le 0.5} \hfill \\ { - 1} \hfill & {Else} \hfill \\ \end{array} } \right.$$

25 $$\ddot{\ell}=\left(1-\overrightarrow{U}\right)\times \overrightarrow{\mathcal{M}}\times \mathcal{L}$$

26 $$\overrightarrow{\mathcal{M}}=\left({r}_{3}\times \left(1-\overrightarrow{{r}_{5}}\right)+\overrightarrow{{r}_{5}}\right),$$

27 $$\overrightarrow{U}_{1}=\left\{ {\begin{array}{*{20}l} 0 \hfill & {\overrightarrow {{r_{5} }} \le {{r_{4} }} } \hfill \\ 1 \hfill & {Else} \hfill \\ \end{array} } \right.,$$

28 $${U}_{2}=\left\{ {\begin{array}{*{20}l} 0 \hfill & {{r_{3}} \le {{r_{4} }} } \hfill \\ 1 \hfill & {Else} \hfill \\ \end{array} } \right.,$$

where $\overrightarrow{{V}_{i}}\left(t\right)$ stands for the $ith$ object’s velocity, ${r}_{3}$ and ${r}_{4}$ are two numbers selected at random in the range (0, 1); and $\overrightarrow{{r}_{5}}$ and $\overrightarrow{{r}_{6}}$ refer to two vectors, including decimal numbers selected at random in the interval (0, 1). ${\overrightarrow{X}}_{a}$ and ${\overrightarrow{X}}_{b}$ are two solutions picked randomly at random from the current solutions; ${M}_{s}\mathrm{ and }{m}_{i}$ refer to the mass of ${\overrightarrow{X}}_{S}\mathrm{ and} {\overrightarrow{X}}_{i}$, respectively; and ${a}_{i}$ is the semimajor axis of the $ith$ object’s elliptical orbit. $\mathcal{F}$ includes either 1 or − 1, selected at random, to change the search direction. ${a}_{i}$ is defined by:

29 $${a}_{i}\left(t\right)={r}_{3}{\times \left[{T}_{i}^{2}\times \frac{\mu (t)\times \left({M}_{s}+{m}_{i}\right)}{4{\pi }^{2}}\right]}^\frac{1}{3}.$$

**Step 4: Escaping from the local optimum**

Most objects in the solar system rotate on their axes and orbit anticlockwise around the Sun; however, there are a few exceptions. This behavior is exploited by KOA in order to break out of local optimal zones by switching the search direction at regular intervals with the help of a flag *F*. This gives the agents a better chance of efficiently searching the entire space.

**Step 5: Updating objects’ positions**

The planets travel in their elliptical orbits around the Sun. Those planets get closer to the Sun for a while and then away from it during rotation. KOA simulates this behavior in two stages: exploration and exploitation. The exploration operator is simulated in KOA when the Planets are far from the Sun, while the exploitation operator is achieved when the planets are closer to the Sun. In KOA, this behavior is mathematically defined as follows:

30 $${\overrightarrow{X}}_{i}\left(t+1\right)={\overrightarrow{X}}_{i}\left(t\right)+\mathcal{F}\times {\overrightarrow{V}}_{i}\left(t\right)+\left({{F}_{g}}_{i}\left(t\right)+\left|r\right|\right)\times \overrightarrow{U}\times \left({\overrightarrow{X}}_{S}\left(t\right)-{\overrightarrow{X}}_{i}\left(t\right)\right).$$

Equation (30) simulates the Sun's gravitational force on the planets, where this equation uses an additional step size based on calculating the distance between the Sun and the current planet multiplied by the gravitational force of the Sun to assist KOA in exploring the regions surrounding the best-so-far solution and finding better solutions in fewer function evaluations. According to^46^, when a planet is distant from the Sun, its velocity will often represent the exploration operator of KOA. However, the Sun's gravitational pull affects this velocity, allowing the current planet to marginally exploit regions near the best solution. Meanwhile, as a planet gets closer to the Sun, its velocity skyrockets, allowing it to escape the Sun's gravitational pull. If the best-so-far solution, referred to as the sun, is local minima, velocity represents local optimal avoidance, and the Sun's gravitational pull is the exploitation operator to aid KOA in assaulting the best-so-far solution to find better solutions. Increasing the eccentricity of a planet’s orbit increases the strength of the gravitational pull between the planet and the sun when a planet approaches the point closest to the sun. On the contrary, when the planet gets away from the sun, the gravitational pull is gradually weakened. When the eccentricity approaches 0, the gravitational pull is minimized because the orbit will be gradually converted from an elliptical shape into a circle shape, and hence all the points on this orbit might approximately have the same gravitational force. More details for this step are presented in the original reference for KOA^46^.

**Step 6: Updating distance with the Sun**

In an additional effort to improve the KOA's search capabilities, the natural variation in the distance to the Sun and planets over time was simulated. When planets are closer to the Sun, the exploitation operator is activated to enhance the convergence rate, whereas when the Sun is distant, the exploration operator is activated to diminish getting stuck in local optima. This principle is imitated using the controlling parameter $h$, which varies progressively over time. If this parameter is large, the exploration operator is used to broaden the distance between the planets' orbits and the Sun; otherwise, the exploitation operator is utilized to maximize the reward from regions surrounding the best-to-date solution. This principle’s mathematical model could be defined by:

31 $${\overrightarrow{X}}_{i}\left(t+1\right)={\overrightarrow{X}}_{i}\left(t\right)\times {\overrightarrow{U}}_{1}+\left(1-{\overrightarrow{U}}_{1}\right)\times \left(\frac{{\overrightarrow{X}}_{i}\left(t\right)+{\overrightarrow{X}}_{S}+{\overrightarrow{X}}_{a}\left(t\right)}{3.0}+h\times \left(\frac{{\overrightarrow{X}}_{i}\left(t\right)+{\overrightarrow{X}}_{S}+{\overrightarrow{X}}_{a}\left(t\right)}{3.0}-{\overrightarrow{X}}_{b}\left(t\right)\right)\right),$$

32 $$h=\frac{1}{{e}^{\eta r}},$$

where $r$ is a number selected randomly in the range (0, 1), while $\eta$ is defined as follows:

33 $$\eta =\left({a}_{2}-1\right)\times {r}_{4}+1,$$

where ${a}_{2}$ represents a cyclic controlling parameter and could be defined as follows:

34 $${a}_{2}=-1-\left(\frac{t\%\frac{{T}_{max}}{\overline{TC}}}{\frac{{T }_{max}}{TC}}\right),$$

where $TC$ represents the number of cycles, and $\%$ is the modulo operator.

**Step 7: Elitism**

This step enacts an elitist strategy to guarantee that planets and the Sun are always in the local-best positions obtained even now, as defined in the following mathematical formula:

35 $${\overrightarrow{X}}_{i, new}(t+1)=\left\{\begin{array}{ll}{\overrightarrow{X}}_{i}(t+1), &\quad if\,f({\overrightarrow{X}}_{i}(t+1)\le f({\overrightarrow{X}}_{i}(t))\\ {\overrightarrow{X}}_{i}\left(t\right), &\quad Else\end{array}.\right.$$

## Proposed algorithm

The accurate estimation of PV parameters is essential for precise modeling, assessment, and control of PV systems. By knowing the near-optimal values of the PV parameters, the performance, efficiency, and reliability of PV systems at different operating conditions can be significantly optimized^49,50^. Some of the practical benefits that can be obtained from the proper estimation of PV parameters are as follows^49,51^:

- Improved design and sizing of PV systems, such as selecting the optimal number and configuration of PV modules, batteries, inverters, etc.
- Improved MPPT algorithms, which can modify the PV system's operating point to extract the highest power output under any given scenario.
- Improved fault diagnosis and detection of PV systems, such as identifying and pinpointing the sources of power losses, degradation, or damage in PV modules or components.
- More accurate prediction and simulation of the PV system behavior, such as estimating the energy yield, environmental impact, power quality, etc.

Therefore, this paper designs a new optimization technique based on integrating the recently presented KOA with two effective mechanisms for accurate estimation of PV parameters for precise modeling, assessment, and control of PV systems. Those mechanisms are used to enhance the exploration and exploitation capabilities of KOA for accurately solving this complicated optimization problem. Generally, in this section, the different steps of KOA, which are represented in initialization, evaluation, ranking-based update mechanism, exploitation improvement mechanism, and KOA’s pseudocode, are extensively described.

### Initialization

Before starting the optimization process, the proposed algorithm creates *N* solutions with nine, five, or seven unknown parameters according to the tackled PV model. These solutions are then initialized at random within the search boundary of each parameter, as described in Eq. (8). The search limit of each unidentified parameter is described in Table 2. These initialized solutions are evaluated according to the objective function discussed later to compute the quality of each solution and extract the solution with the lowest objective value to represent the best-to-date solution.

**Table 2:** Search limit for each unidentified parameter.

	${I}_{ph}(A)$	${I}_{sdi}\left(A\right), i\in 1:3$	${R}_{s}(\Omega )$	${R}_{sh}(\Omega )$	$n1$	$n2$	$n3$
${X}_{i,up}^{j}$	${1.1I}_{SC}$	$10 \mu A$	$0.5$	$500$	$2$	$2$	$2$
${X}_{i,low}^{j}$	${0.9I}_{SC}$	$1 nA$	$0$	$0$	$1$	$1.2$	$1.4$

#### Objective function (OF)

Finding the unknown parameter values that produce the smallest disparity between the measured and simulated current data is a primary goal when solving the PV models’ parameter identification. Therefore, the RMSE metric between measured and current data is utilized as an OF to determine the quality of the parameters obtained by each solution in the hope of finding the near-optimal solution, which could minimize RMSE as small as possible. The OF’s mathematical formula is as follows:

36 $$RMSE=f\left({\overrightarrow{X}}_{i}\right)= \sqrt{\frac{1}{M}*{\sum_{k=1}^{M}{\left({I}_{m}-I\left({V}_{e}, {\overrightarrow{X}}_{i}\right)\right)}^{2}}},$$

where $I$ and ${I}_{m}$ stand for the estimated current and the measured current, respectively. $M$ refers to the number of measured data points. ${\overrightarrow{X}}_{i}$ contains the values of the unidentified parameters obtained by the $ith$ solution. For each solution in the population, the estimated current $I\left({V}_{e}, {\overrightarrow{X}}_{i}\right)$ is computed by the Newton–Raphson method for each set of experimentally-measured points^52^.

### Ranking-based update mechanism (RUM)

In^53^, a new mechanism called RUM was proposed to weed out the solutions that could not achieve better solutions in a number of successive iterations, and replace them with new solutions generated using some of the updating schemes. Those schemes must be designed in a manner that aids in covering the regions intractable to a metaheuristic algorithm. The main disadvantage of this mechanism is that no specific updating scheme could be used to generate new solutions. Although this is considered a disadvantage, it is also considered an advantage at the same time because it gives the researchers more flexibility in finding the most relevant schemes that could enhance performance. In^53^, the author employed an updating scheme that aimed to speed up convergence to the best-to-date solution by shifting the less-beneficial solutions to the region between the best solution obtained yet and the current position. This was done in the hope that a better solution could be found in this region. However, this scheme might lead to falling into local optima when handling the problem considered in this study, which needs strong exploratory patterns to be accurately solved. Therefore, in this study, a new updating mechanism is designed to help enhance the convergence speed in addition to diminishing stagnation in local optima. This mechanism is based on two folds. The first fold is based on borrowing the fish aggregating devices (FADs) from MPA to promote the exploration operator. FADs, according to^54^, are mathematically represented in the following formula:

37 $${\overrightarrow{{\text{X}}}}_{{\text{i}}}=\left\{\begin{array}{ll}{\overrightarrow{{\text{X}}}}_{{\text{i}}}+CF*\left[{\overrightarrow{X}}_{i,low}+{r}_{2}*\left({\overrightarrow{X}}_{i,up}- {\overrightarrow{X}}_{i,low}\right)\right]\otimes \overrightarrow{{\text{U}}} &\quad if\,r<FADs\\ {\overrightarrow{{\text{X}}}}_{{\text{i}}}+\left[FADs*\left(1-{\text{r}}\right)+{\text{r}}\right]\left({\overrightarrow{{\text{X}}}}_{{\text{r}}1}-{\overrightarrow{{\text{X}}}}_{{\text{r}}2}\right) &\quad Else\end{array},\right.$$

where ${\overrightarrow{{\text{X}}}}_{{\text{r}}1}$ and ${\overrightarrow{{\text{X}}}}_{{\text{r}}2}$ are two individuals chosen at random from the current individuals, ${{\text{r}}}_{2}$ is the numerical value selected randomly in (0, 1), $FADs$ was set to 0.2 in MPA, but here is generated randomly between 0 and 1 to avoid parameter tuning burden, $\otimes$ is element-wise multiplication operator, $\overrightarrow{{\text{U}}}$ is a binary vector used to determine whether each dimension in the current solution is updated or not, and ${\text{CF}}$ is an adaptive controlling parameter, which is regenerated in each generation by:

38 $$CF={(1-\frac{t}{{T}_{max}})}^{\left(2\frac{t}{{T}_{max}}\right)}.$$

The second fold is based on designing updating schemes capable of further exploring the search space for reaching promising regions, which might contain the desired solution. Those updating schemes were based on the levy flight and normal distribution to give variety in the generated step sizes for covering the search space as much as possible. The second fold is mathematically formulated as follows:

39 $${\overrightarrow{{\text{X}}}}_{{\text{i}}}=\left\{\begin{array}{ll}{\overrightarrow{{\text{X}}}}_{{\text{i}}}+RL*\left({\text{RL}}*{\overrightarrow{X}}_{S}\left(t\right)-{\overrightarrow{{\text{X}}}}_{{\text{i}}}\right) &\quad if\,r<{r}_{2}\\ {\overrightarrow{{\text{X}}}}_{{\text{a}}}+rn*\left({\text{rn}}*{\overrightarrow{X}}_{b}\left(t\right)-{\overrightarrow{{\text{X}}}}_{{\text{r}}1}\right) &\quad Else\end{array}\right.,$$

where ${\text{RL}}$ is a numerical value generated according to the levy flight. The tradeoff between the first and second fold within our proposed algorithm is achieved at random, as defined in the following equation:

40 $${\overrightarrow{{\text{X}}}}_{{\text{i}}} = \left\{ {\begin{array}{*{20}l} Eq. (37) \hfill & if\,{{r}} \le {{r_{2}}} \hfill \\ Eq. (39) \hfill & {Else} \hfill \\ \end{array} } \right.$$

Before starting the optimization process, a variable is generated for each solution to contain the number of successive times it could not achieve a better solution. In the case that the variable of a solution contains a number greater than the threshold value, this solution will be replaced with a new solution generated under Eq. (40).

### Exploitation improvement strategy

The method discussed in the previous section is integrated into KOA to further improve its exploration pattern, but its exploitation operator still needs further improvement to achieve better solutions in lower function evaluations. Therefore, in this paper, an additional improvement strategy, namely the exploitation improvement strategy, is proposed to relate the updating process of the current solution with the best solution achieved yet for searching for a better solution as quickly as possible. This strategy’s mathematical model is presented in the following formula:

41 $${\overrightarrow{{\text{X}}}}_{{\text{i}}}=\left\{\begin{array}{ll}{\overrightarrow{{\text{X}}}}_{{\text{i}}}\otimes \overrightarrow{{{\text{U}}}_{1}}+\left(\frac{\left({\overrightarrow{{\text{X}}}}_{{\text{i}}}+{\overrightarrow{X}}_{S}\left(t\right)\right)}{2}+\left|{\text{rn}}\right|*\left(\frac{\left({\overrightarrow{{\text{X}}}}_{{\text{i}}}+{\overrightarrow{X}}_{S}\left(t\right)\right)}{2}-{\overrightarrow{{\text{X}}}}_{{\text{i}}}\right)\right)\otimes \left(1-\overrightarrow{{{\text{U}}}_{1}}\right) &\quad if\,r<{r}_{2}\\ {\overrightarrow{X}}_{S}\left(t\right)+{{\text{r}}}_{1}*\left({\overrightarrow{X}}_{S}\left(t\right)-{\overrightarrow{X}}_{i}\left(t\right)\right)+(1-{{\text{r}}}_{1})*\left({\overrightarrow{X}}_{a}\left(t\right)-{\overrightarrow{X}}_{b}\left(t\right)\right) &\quad Else\end{array}\right.,$$

where ${{\text{r}}}_{1}$ is a number selected at random in (0, 1), and $\overrightarrow{{{\text{U}}}_{1}}$ is a binary vector assigned with 1 and 0 according to a convergence rate (CR) factor estimated in the experiments section to prevent premature convergence that might be caused by the first state in the previous equation. In brief, $\overrightarrow{{{\text{U}}}_{1}}$ is mathematically defined as follows:

42 $$\overrightarrow{{{\text{U}}}_{1}}=\left\{\begin{array}{ll}1 &\quad i f \overrightarrow{{\text{R}}}<CR\\ 0 &\quad Else\end{array}\right.,$$

where $\overrightarrow{{\text{R}}}$ is a vector including numbers generated at random between 0 and 1. Finally, this strategy and RUM are integrated with the classical KOA in an effective manner to enhance its search potential. In a more sense, the classical KOA is extensively applied at the beginning of the optimization process for covering all the possible regions within the search space that might involve the desired solution. In addition, to further improve the KOA’s exploration operator, the RUM is applied to replace the solutions of KOA that could not achieve better solutions for RK successive iterations. With increasing the current function evaluation, HKOA increases the probability of the exploitation improvement strategy to extensively exploit the regions around the best solution achieved yet for finding the required solution. This optimization procedure is repeated until the termination criterion is met. In brief, the pseudocode of HKOA is listed in Algorithm 1.

**Algorithm 1:**
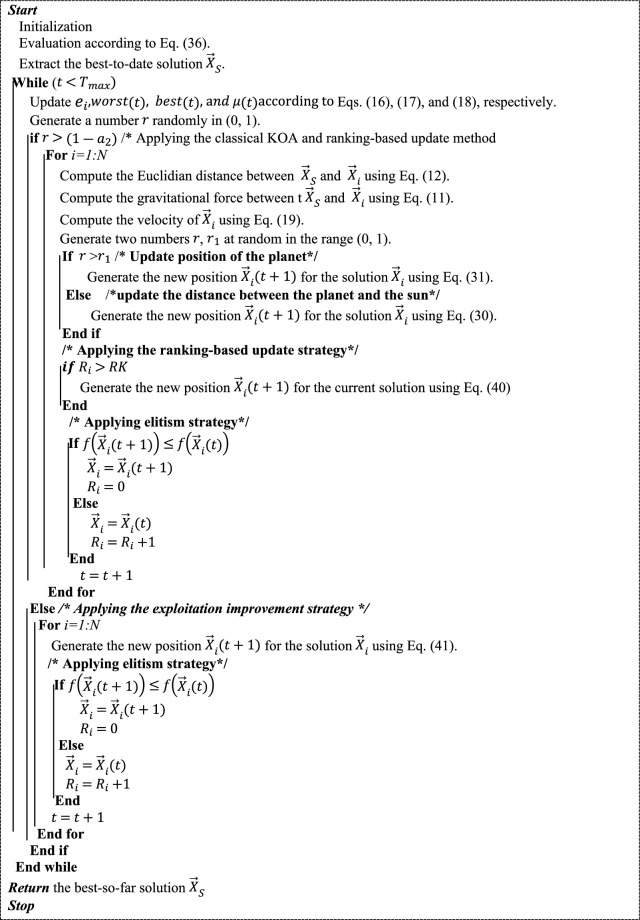
Pseudo-code of HKOA.

## Results and discussion

This section is presented to assess the HKOA performance for the parameter identification of three PV models for a solar cell called RTC France and five PV modules, including Ultra, PWP, STP6, KKC, and STM. All those PV modules are based on three different PV models: SDM, DDM, and TDM, to investigate the accuracy of the proposed HKOA for estimating the unknown parameters for them. Those PV modules are used in our experiments due to their wide use in the literature and their different characteristics that aid in discovering the stability of the newly proposed algorithms^55,56^. Those characteristics at STC are the number of cells connected in series in PV modules (${N}_{s}$), the open-circuit current–voltage (${V}_{oc}$), the maximum output current (${I}_{m}$), the maximum output power (${P}_{m}$), the short-circuit current point (${I}_{SC}$), the maximum output voltage (${V}_{m}$), the short-circuit current–temperature factor(${k}_{i}$), and the temperature coefficient of open-circuit voltage (${k}_{v}$) and are set in our experiments as reported in Table 2^57^.

In our conducted experiments, HKOA was executed 30 independent times on each module to remedy its stochastic nature, and its outcomes are analyzed in terms of several performance metrics, such as standard deviation (SD), best RMSE, average (Avg) RMSE, and Worst RMSE. In addition, the convergence curve is used in the comparison to disclose the convergence speed of each algorithm; the Wilcoxon rank-sum test is used to show the difference between each pair of algorithms; and the computational cost is used to compute the time consumed by each algorithm until completing the optimization process. To reveal the HKOA’s effectiveness, its values for various performance metrics were contrasted with three different categories of optimizers: the first category includes some recently-published metaheuristic techniques, such as pelican optimization algorithm (POA)^58^, gorilla troops optimizer (GTO)^59^, dandelion optimizer (DO)^60^, and classical KOA^46^; the second category includes two high-performing optimization techniques, such as particle swarm optimization (PSO)^61^ and LSHADE_cnEpSin (cnEpSin)^62^; the last category includes some of the high-performing optimization techniques proposed recently for tackling the parameter estimation problem of PV models, including ranking-based whale optimization algorithm (RWOA)^55^, interior search algorithm (ISA)^63^, and spider wasp optimizer (SWO)^64^. The controlling parameters of those rival optimizers were set as suggested in the cited paper, except for ${T}_{max}$ and $N$, which are set to 25,000 and 25, respectively, to guarantee a fair comparison. The search limit of each unidentified parameter in various PV models is set as stated in Table 3. Ultimately, the experiments in this section were conducted on a computer that possessed the following attributes: 32GB RAM, 2.40GHz Intel(R) Core(TM) i7-4700MQ processor, 64-bit Windows 10 Professional. All algorithms are implemented in MATLAB R2019a.

**Table 3:** Characteristics of the RTC France solar cell and PV modules.

Characteristics	KKC	PWP	RTC	STP	Ultra	STM
${P}_{m}[W]$	200	11.5	0.31	102	85	25.5
${V}_{m}[V]$	26.3	12.649	0.459	14.93	17.2	16.98
${I}_{m}[A]$	7.61	0.912	0.6755	6.83	4.95	1.5
${V}_{oc}[V]$	32.9	16.7785	0.5736	19.21	22.2	21.02
${I}_{SC}[A]$	8.21	1.0317	0.7605	7.48	5.45	1.663
${N}_{s}$	54	36	1	36	36	36
${K}_{i}$	0.0318	0.0008	0.000387	0.00065	0.0008	− 0.00065
${K}_{v}$	− 0.123	− 0.0725	− 0.003739	− 0.003466	− 0.0725	− 0.00346

### RTC France solar cell

The unknown parameters of DDM, TDM, and SDM based on the RTC France solar cell are estimated in this section using HKOA to disclose its effectiveness. Information on current and voltage was collected from a commercial silicon RTC France solar cell of 57 mm diameter and operating at a temperature of 33 °C ^64^.

A. Single-Diode model

**Figure 5:**
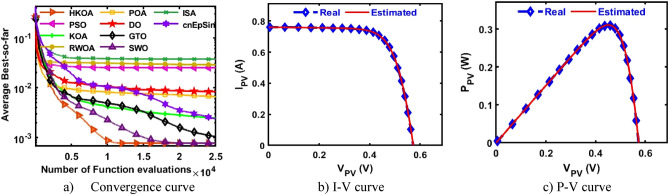
Comparison among algorithms under RTC France cell based on SDM.

Table 4 reports the results of various performance metrics after each optimizer was run 30 times on the SDM-based RTC France. This table shows that HKOA is comparable to SWO and better than the others for all performance metrics. The WRS test is also utilized to show that there is a difference between the HKOA’s findings and those of the other algorithms. For each pair of algorithms, the WRS test provides a number called a p-value, which indicates whether there is a difference between each pair of algorithms or not. The p-value of HKOA against each algorithm is reported in Table 4, which shows that HKOA is substantially different from all competitors except SWO. Since HKOA is competitive with SWO in terms of the majority of performance metrics and the WRS test, an additional performance indicator called the convergence curve is used to disclose the HKOA's ability to reach the near-optimal solution faster than the others. This indicator for each algorithm within the whole optimization process is computed and reported in Fig. 5a. Inspecting this figure reveals that HKOA is the best algorithm, where it could reach the smallest RMSE after 10,000, while SWO, which is considered the second-best algorithm, needs around 15,000 to reach the smallest RMSE. This superiority is due to the exploitation improvement mechanism that aids in exploiting the regions around the best-so-far solution, thereby aiding in accelerating the convergence speed of HKOA in comparison to KOA and all compared algorithms. Additionally, P–V and I-V curves based on the best parameters obtained by HKOA are depicted in Fig. 5b,c to show the consistency between the estimated and measured current. Those figures show that the unknown parameters estimated by HKOA could reach current and power that are highly consistent with the measured.

**Table 4:** Comparison among algorithms using RTC France cell under SDM.

	Best-obtained parameters					RMSE				*P* value
	${I}_{ph}(A)$	${I}_{sd}(\mu A)$	${R}_{s}(\Omega )$	${R}_{sh}(\Omega )$	$n$	$Best$	*Worst*	*Avg*	*SD*	
HKOA	**0.7608**	**3.107E-07**	**0.0365**	**52.8898**	**1.4773**	**7.73006E-04**	**7.73006E-04**	**7.73006E-04**	**4.04037E-17**	
PSO	**0.7608**	**3.107E-07**	**0.0365**	**52.8898**	**1.4773**	**7.73006E-04**	7.36945E-02	2.51591E-02	3.49069E-02	**5.57E-07**
KOA	0.7603	7.155E-07	0.0327	83.4305	1.5660	1.56653E-03	3.69726E-03	2.45608E-03	5.57207E-04	**3.02E-11**
RWOA	0.7606	6.282E-07	0.0334	79.0325	1.5513	1.37972E-03	7.36945E-02	2.87527E-02	3.47952E-02	**3.00E-11**
POA	0.7624	1.498E-06	0.0287	62.0875	1.6544	3.25924E-03	8.78483E-03	6.56213E-03	1.48056E-03	**3.02E-11**
DO	0.7607	5.819E-07	0.0337	68.7958	1.5430	1.27482E-03	7.36945E-02	8.33639E-03	1.26479E-02	**3.02E-11**
GTO	0.7608	3.101E-07	0.0366	52.8539	1.4771	7.73012E-04	1.48373E-03	1.04463E-03	2.20310E-04	**3.02E-11**
SWO	**0.7608**	**3.107E-07**	**0.0365**	**52.8898**	**1.4773**	**7.73006E-04**	**7.73006E-04**	**7.73006E-04**	4.76558E-16	0.976411
ISA	0.7606	3.369E-07	0.0362	56.2926	1.4854	7.89315E-04	7.36945E-02	3.75144E-02	3.67999E-02	**2.70E-11**
cnEpSin	0.7608	3.107E-07	0.0365	52.8898	1.4773	7.73006E-04	6.86335E-03	2.39681E-03	1.89506E-03	**3.65E-08**

Bold font represents the best outcomes.

B. Double-Diode model

**Figure 6:**
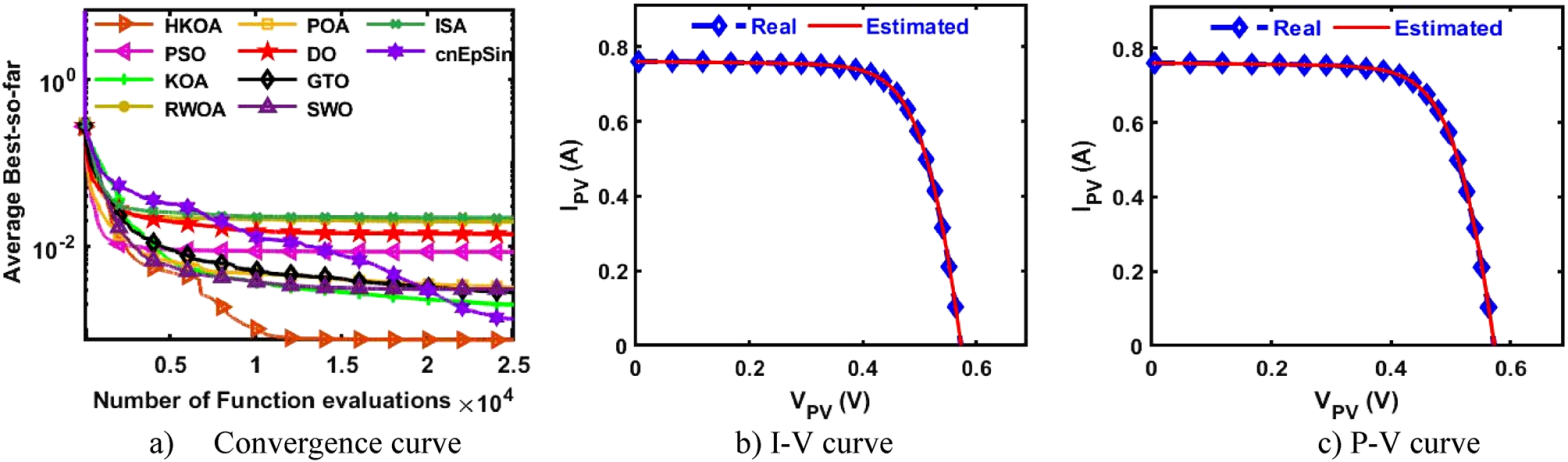
Comparison among algorithms under RTC France cell based on DDM.

After each method has been executed thirty times on the DDM-based RTC France, various performance indicators are calculated and provided in Table 5. This table shows that HKOA achieves the highest ranking, followed by cnEpSin in second place, while ISA is considered the worst-performing algorithm. The p-value, which is also included in this table, demonstrates that there is a substantial difference between the outcomes of HKOA and those of its competitors. Also, Fig. 6a depicts the convergence rate for each algorithm to further demonstrate the HKOA’s superiority. From this figure, HKOA could achieve the smallest RMSE after around 10,000 function evaluations, while the compared algorithms until completing the maximum number of function evaluations could not reach a smaller RMSE value than that achieved by HKOA. Therefore, HKOA is considered faster and better than all the compared algorithms when applied to estimate the unknown parameters of the DDM-based RTC France solar cell. Figure 6b,c show that the P–V and I-V estimated by the parameters of HKOA are highly consistent with the measured data.

**Table 5:** Comparison between HKOA and its competitors using RTC France cell under DDM.

	Best-obtained parameters							RMSE				p-value
	${I}_{ph}(A)$	${I}_{sd1}(A)$	${I}_{sd2}(A)$	${R}_{s}(\Omega )$	${R}_{sh}(\Omega )$	${{\text{n}}}_{1}$	${{\text{n}}}_{2}$	Best	Worst	Avg	SD	
HKOA	**0.761**	**8.66E-08**	**2.16E-06**	**0.038**	**58.356**	**1.373**	**2.000**	**7.326E-04**	**7.730E-04**	**7.473E-04**	**1.232E-05**	
PSO	0.761	9.42E-08	1.31E-06	0.038	56.724	1.382	1.893	7.383E-04	6.944E-02	8.506E-03	2.068E-02	**5.00E-09**
KOA	0.760	2.54E-07	7.77E-07	0.036	66.412	1.466	1.906	9.392E-04	3.059E-03	1.984E-03	5.335E-04	**3.02E-11**
RWOA	0.761	7.14E-08	2.42E-06	0.038	59.502	1.358	2.000	7.338E-04	6.944E-02	1.951E-02	3.063E-02	**6.47E-08**
POA	0.761	1.60E-07	1.49E-06	0.037	63.298	1.423	2.000	7.869E-04	7.756E-03	3.159E-03	1.846E-03	**3.02E-11**
DO	0.760	4.37E-09	4.99E-06	0.041	125.281	1.170	2.000	1.140E-03	6.944E-02	1.389E-02	2.529E-02	**3.02E-11**
GTO	0.761	2.37E-07	4.35E-07	0.036	55.888	1.457	1.868	7.712E-04	5.842E-03	2.763E-03	1.291E-03	**3.34E-11**
SWO	0.761	9.98E-08	1.89E-06	0.038	57.607	1.384	1.990	7.336E-04	6.944E-02	3.046E-03	1.254E-02	**2.50E-03**
ISA	0.761	1.95E-07	9.37E-07	0.037	55.147	1.438	2.000	7.490E-04	6.944E-02	2.188E-02	3.167E-02	**9.85E-11**
cnEpSin	0.761	1.84E-07	1.02E-06	0.037	55.093	1.433	1.999	7.468E-04	6.467E-03	1.305E-03	1.418E-03	**4.94E-05**

Bold font represents the best outcomes.

B. Triple-Diode model

**Figure 7:**
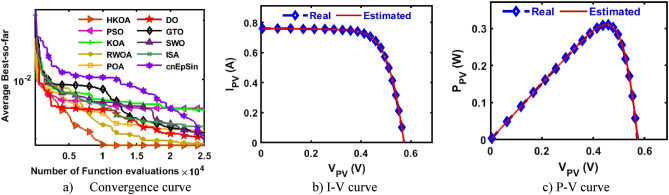
Comparison among algorithms under RTC France cell based on TDM.

In this section, HKOA is applied to identify nine unknown parameters of TDM based on RTC France to further reveal its performance. Table 6 shows the outcomes obtained by various algorithms for this case. This table shows that HKOA is the best-performing algorithm, SWO is the second-best algorithm, and DO is the worst. This table also includes the p-value between HKOA and each rival optimizer; this value indicates that HKOA results are different from those of all other algorithms. The convergence curve for each algorithm is illustrated in Fig. 7a to further illustrate HKOA's superiority. According to this figure, HKOA was able to obtain the lowest RMSE after about 10,000 function evaluations, meanwhile, all rival algorithms were unable to acquire a lower RMSE value than HKOA up until they finished the maximum number of function evaluations. As a result, when used to estimate the unknown parameters of the TDM-based RTC France solar cell, HKOA is thought to be quicker and more accurate than all of the compared algorithms. In addition, Fig. 7b,c are presented to show consistency between the I-V and P–V curves estimated by HKOA against the measured curves. Those figures show that the estimated data is highly identical to the measured data.

**Table 6:** Comparison among algorithms using RTC France cell under TDM.

	Best-obtained parameters									RMSE				*P* value
	${I}_{ph}(A)$	${I}_{sd1}(A)$	${I}_{sd2}(A)$	${I}_{sd3}(A)$	${R}_{s}(\Omega )$	${R}_{sh}(\Omega )$	${{\text{n}}}_{1}$	${{\text{n}}}_{2}$	${{\text{n}}}_{3}$	$Best$	Worst	Avg	SD	
HKOA	**0.760**	**2.0E-09**	**1.0E-07**	**2.0E-06**	**0.038**	**61.021**	**61.02**	**1.32**	**1.39**	**7.511E-04**	**7.87E-04**	**7.64E-04**	**9.9E-06**	
PSO	0.760	9.2E-08	3.8E-07	1.6E-06	0.038	60.977	60.98	1.38	1.98	7.514E-04	2.87E-03	1.24E-03	6.6E-04	**2.E-05**
KOA	0.759	3.0E-07	2.6E-07	3.7E-07	0.037	131.380	131.38	1.89	1.47	1.701E-03	4.59E-03	3.06E-03	7.9E-04	**3.E-11**
RWOA	0.761	7.5E-08	2.4E-06	1.0E-09	0.038	62.624	62.62	1.36	2.00	7.518E-04	9.99E-04	8.29E-04	7.3E-05	**7.E-06**
POA	0.761	9.3E-09	9.7E-07	1.5E-07	0.039	59.736	59.74	1.23	1.71	7.793E-04	5.71E-03	2.47E-03	1.0E-03	**4.E-11**
DO	0.760	1.0E-09	5.4E-06	3.5E-09	0.043	149.038	149.04	1.09	2.00	1.284E-03	5.47E-03	3.32E-03	1.1E-03	**3.E-11**
GTO	0.760	8.4E-07	1.4E-07	1.0E-09	0.037	58.256	58.26	1.86	1.42	7.641E-04	4.43E-03	1.46E-03	8.0E-04	**3.E-10**
SWO	0.760	7.1E-08	2.4E-06	8.9E-09	0.038	62.608	62.61	1.36	2.00	7.520E-04	1.19E-03	7.91E-04	7.7E-05	**2.E-04**
ISA	0.760	1.7E-06	1.6E-09	1.2E-07	0.038	61.218	61.22	2.00	1.94	7.666E-04	2.78E-03	1.41E-03	5.5E-04	**1.E-10**
cnEpSin	0.760	2.3E-07	1.6E-09	2.4E-07	0.037	55.865	55.87	1.79	2.00	7.801E-04	5.62E-03	3.11E-03	1.4E-03	**4.E-11**

Bold font represents the best outcomes.

### PWP module

In this section, the HKOA’s performance is investigated to identify the unknown parameters of three PV models under a well-known PV module, namely the PWP module.

A. Single-Diode model

**Figure 8:**
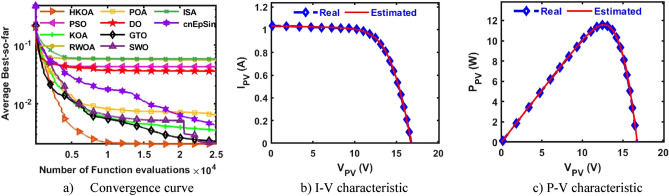
Comparison among algorithms under PWP module based on SDM.

After each algorithm was executed 30 times on the SDM-based PWP, the results of various performance metrics are shown in Table 7. Based on this data, it is clear that HKOA is superior to all the other optimizers for the majority of the performance metrics. To further demonstrate that the HKOA's outcomes are distinct from those of its competitors, we employ the WRS test. Table 7 reports the p-value of the WRS test between HKOA and each rival algorithm. This value demonstrates that, except for SWO, HKOA is substantially different from its competitors. In addition, the convergence curve is presented in Fig. 8a to show how HKOA gets closer to the best-to-date solution in less time. This figure shows that after fewer than 10,000 function evaluations, HKOA was able to obtain the lowest RMSE. In contrast, some competing algorithms, such as GTO and SWO, at the end of the optimization process could approximate slightly the smallest RMSE value obtained by HKOA. Thus, HKOA is considered to be the best when used to estimate the unknown parameters of the SDM-based PWP module. In addition, Fig. 8b,c depict I-V and P–V curves using the best parameters obtained by HKOA, demonstrating consistency between the measured and calculated current.

**Table 7:** Comparison among algorithms using PWP under SDM.

	Best-obtained parameters					RMSE				*P* value
	${I}_{ph}(A)$	${I}_{sd}(\mu A)$	${R}_{s}(\Omega )$	${R}_{sh}(\Omega )$	$n$	$Best$	*Worst*	*Avg*	*SD*	
HKOA	**1.0324**	**2.497E-06**	**0.0345**	**20.7868**	**1.3166**	**2.03999E-03**	**2.03999E-03**	**2.03999E-03**	**1.713E-17**	
PSO	**1.0324**	**2.497E-06**	**0.0345**	**20.7867**	**1.3166**	**2.03999E-03**	9.51542E-02	4.25039E-02	4.683E-02	**5.70E-08**
KOA	1.0314	4.181E-06	0.0328	25.8651	1.3713	2.41293E-03	4.75281E-03	3.49481E-03	6.109E-04	**3.02E-11**
RWOA	1.0311	3.821E-06	0.0331	27.1097	1.3612	2.24311E-03	9.51542E-02	5.53996E-02	4.624E-02	**2.86E-11**
POA	1.0286	5.607E-06	0.0319	53.4566	1.4038	2.80005E-03	1.35118E-02	6.38457E-03	2.462E-03	**3.02E-11**
DO	1.0326	5.264E-06	0.0317	25.0082	1.3972	2.78688E-03	9.51542E-02	3.57308E-02	4.279E-02	**3.02E-11**
GTO	1.0324	2.509E-06	0.0344	20.8080	1.3171	2.04005E-03	3.07165E-03	2.28062E-03	2.993E-04	**3.02E-11**
SWO	**1.0324**	**2.497E-06**	**0.0345**	**20.7867**	**1.3166**	**2.03999E-03**	4.65638E-03	2.12721E-03	4.777E-04	4.25E-01
**ISA**	1.0317	2.752E-06	0.0342	22.5303	1.3266	2.05467E-03	9.51542E-02	5.81411E-02	4.611E-02	**2.25E-11**
**cnEpSin**	**1.0324**	**2.497E-06**	**0.0345**	**20.7867**	**1.3166**	**2.03999E-03**	6.89645E-03	4.22208E-03	1.606E-03	**8.14E-11**

Bold font represents the best outcomes.

B. Double-Diode model

**Figure 9:**
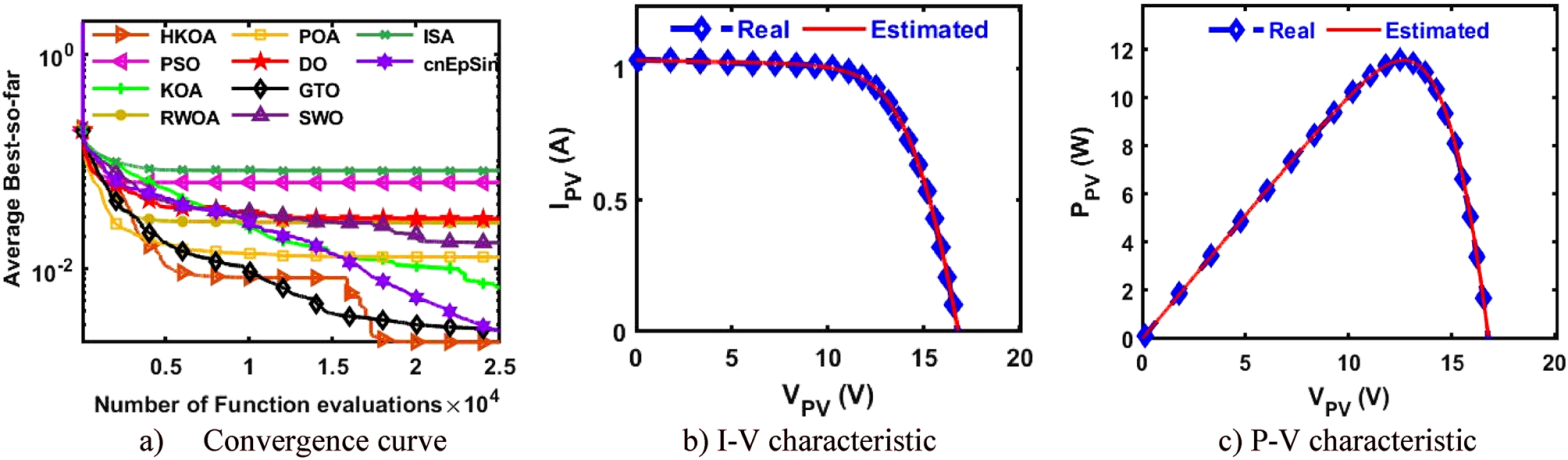
Comparison among algorithms under PWP module based on DDM.

After each method has been executed thirty times on the PWP based on DDM, various performance metrics are computed and displayed in Table 8. Inspecting this table shows that HKOA is the best, followed by cnEpSin in second place, and ISA is the algorithm with the worst performance. This table also includes the p-value, which demonstrates that there is a difference between the outcomes of HKOA and those of its competitors. The convergence curve shown in Fig. 9a further illustrates the superiority of HKOA. In a more general sense, this figure shows that HKOA had a convergence speed competitive with that of GTO until reaching the function evaluation of 15,000. Afterwards, HKOA could converge faster and achieve an RMSE lower than all the compared algorithms. As a result, HKOA is regarded as the best method for estimating the unknown parameters of the DDM-based PWP module. The I-V and P–V estimated by the HKOA parameters are highly consistent with the measured data, as shown in Fig. 9b,c.

**Table 8:** Comparison between HKOA and its competitors using PWP under DDM.

	Best-obtained parameters							RMSE				*P* value
	${I}_{ph}(A)$	${I}_{sd1}(A)$	${I}_{sd2}(A)$	${R}_{s}(\Omega )$	${R}_{sh}(\Omega )$	${{\text{n}}}_{1}$	${{\text{n}}}_{2}$	Best	Worst	Avg	SD	
HKOA	**1.032**	**4.32E-07**	**2.06E-06**	**0.034**	**20.787**	**1.317**	**1.317**	**2.040E-03**	**2.185E-03**	**2.050E-03**	**2.816E-05**	
PSO	1.032	1.35E-08	2.94E-06	0.035	21.493	1.069	1.346	2.086E-03	9.391E-02	6.351E-02	4.373E-02	**5.94E-11**
KOA	1.030	1.74E-06	4.39E-06	0.035	27.667	1.289	1.714	2.364E-03	8.158E-02	6.628E-03	1.432E-02	**3.02E-11**
RWOA	**1.032**	**8.00E-09**	**2.52E-06**	**0.034**	**20.935**	**1.292**	**1.318**	**2.040E-03**	9.390E-02	2.678E-02	4.117E-02	**9.76E-10**
POA	1.032	2.08E-06	4.21E-06	0.035	21.200	1.301	1.932	2.097E-03	9.390E-02	1.270E-02	2.756E-02	**3.34E-11**
DO	1.035	1.00E-09	1.53E-06	0.036	15.441	1.030	1.271	2.334E-03	9.391E-02	2.902E-02	3.985E-02	**3.02E-11**
GTO	1.032	1.20E-06	1.54E-06	0.034	21.652	1.299	1.356	2.050E-03	5.057E-03	2.665E-03	6.855E-04	**1.61E-10**
SWO	**1.032**	**1.00E-09**	**2.50E-06**	**0.034**	**20.787**	**1.312**	**1.317**	**2.040E-03**	9.390E-02	1.744E-02	3.478E-02	**2.13E-05**
ISA	1.032	1.74E-06	4.83E-06	0.035	21.271	1.286	1.826	2.124E-03	9.390E-02	8.173E-02	3.157E-02	**3.69E-11**
cnEpSin	**1.032**	**6.12E-07**	**1.91E-06**	**0.034**	**20.946**	**1.293**	**1.327**	**2.042E-03**	3.726E-03	2.610E-03	4.493E-04	**3.16E-10**

Bold font represents the best outcomes.

C. Triple-Diode model

**Figure 10:**
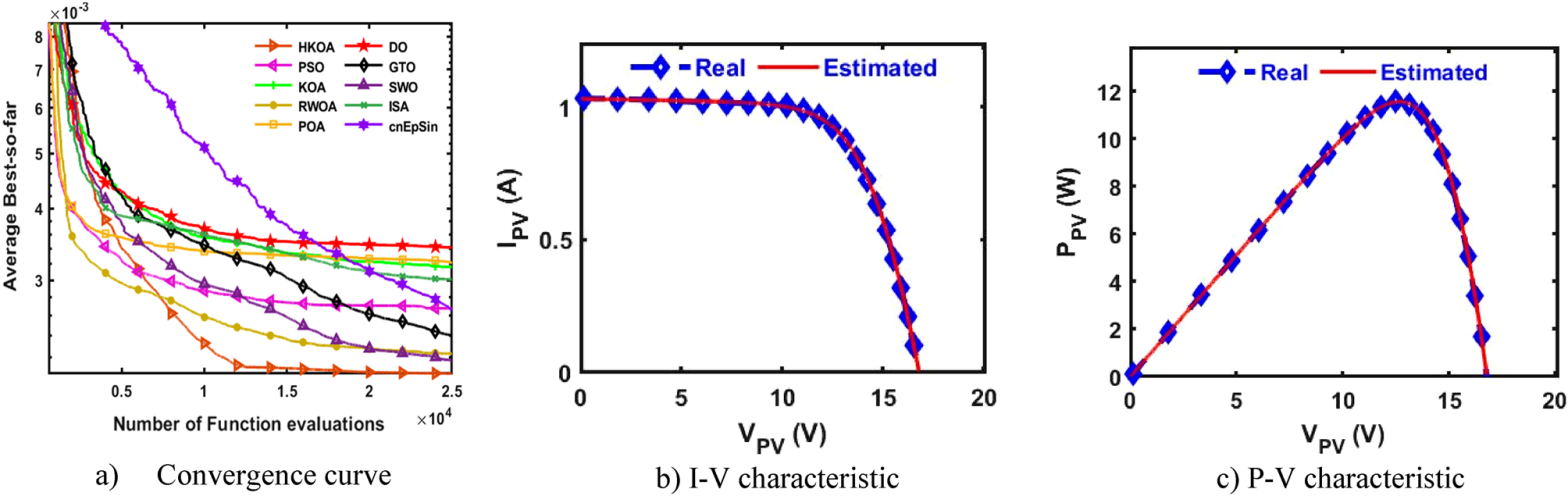
Comparison among algorithms under PWP module based on TDM.

In this section, HKOA is utilized to estimate nine unidentified TDM parameters based on the PWP module in order to disclose more about its performance. Table 9 demonstrates the results acquired by various algorithms in this scenario. Based on this table, HKOA is the algorithm with the greatest performance, followed by SWO in second place and DO in last place. This table also includes the p-value between HKOA and each algorithm compared; this value indicates that the results of HKOA are distinct from those of all other algorithms. Figure 10a depicts the convergence curves for each algorithm to further demonstrate HKOA's superiority. This figure shows that after around 12,000 function evaluations, HKOA was able to obtain the lowest RMSE. In contrast, all competing algorithms were unable to obtain a lower RMSE value than HKOA even after completing the maximum number of function evaluations. Thus, HKOA is considered to be faster and more accurate than all of the analyzed algorithms when used to estimate the unknown parameters of the TDM-based PWP module. Moreover, Fig. 10b,c are provided to illustrate the consistency between the I-V and P–V curves estimated by HKOA and the measured curves. These figures demonstrate that the anticipated and measured data are extremely similar.

**Table 9:** Comparison among algorithms using PWP under TDM.

	Best-obtained parameters									RMSE				*P* value
	${I}_{ph}(A)$	${I}_{sd1}(A)$	${I}_{sd2}(A)$	${I}_{sd3}(A)$	${R}_{s}(\Omega )$	${R}_{sh}(\Omega )$	${{\text{n}}}_{1}$	${{\text{n}}}_{2}$	${{\text{n}}}_{3}$	$Best$	Worst	Avg	SD	
HKOA	**1.032**	**2.3E-06**	**3.2E-07**	**1.0E-09**	**0.034**	**22.16**	**1.32**	**1.32**	**1.40**	**2.051E-03**	**2.14E-03**	**2.06E-03**	**2.1E-05**	
PSO	**1.032**	**1.0E-09**	**2.6E-06**	**1.9E-08**	**0.034**	**22.17**	**1.65**	**1.32**	**1.40**	**2.051E-03**	3.58E-03	2.68E-03	5.2E-04	**2.4E-09**
KOA	1.028	4.4E-06	3.6E-06	1.5E-07	0.033	61.32	1.98	1.36	1.65	2.817E-03	3.60E-03	3.16E-03	2.1E-04	**3.0E-11**
RWOA	**1.032**	**1.0E-09**	**2.6E-06**	**1.1E-09**	**0.034**	**22.29**	**1.34**	**1.32**	**2.00**	**2.051E-03**	2.67E-03	2.23E-03	1.9E-04	**4.6E-09**
POA	1.032	5.8E-07	2.9E-06	1.0E-09	0.034	23.14	1.98	1.33	1.40	2.079E-03	4.20E-03	3.22E-03	4.4E-04	**5.0E-11**
DO	1.030	3.0E-06	8.3E-06	1.3E-07	0.033	36.00	1.34	2.00	1.91	2.413E-03	4.30E-03	3.42E-03	4.0E-04	**3.0E-11**
GTO	1.032	1.0E-09	2.8E-06	1.7E-08	0.034	22.74	1.81	1.33	1.46	2.072E-03	3.27E-03	2.40E-03	3.2E-04	**1.6E-10**
SWO	**1.032**	**9.9E-07**	**1.6E-06**	**1.0E-09**	**0.034**	**22.16**	**1.32**	**1.32**	**1.71**	**2.051E-03**	3.35E-03	2.17E-03	2.9E-04	**1.0E-04**
ISA	1.031	1.0E-09	2.7E-06	1.6E-09	0.034	23.41	1.66	1.32	1.65	2.079E-03	4.06E-03	3.01E-03	5.4E-04	**5.0E-11**
cnEpSin	1.032	1.8E-06	2.9E-06	1.2E-09	0.033	25.03	1.31	1.50	1.60	2.275E-03	2.92E-03	2.66E-03	1.5E-04	**3.0E-11**

Bold font represents the best outcomes.

### STM6-40 module

This section investigates the efficacy of HKOA for identifying the unknown parameters of three PV models under a well-known PV module called the STM module.

A. Single-Diode model

**Figure 11:**
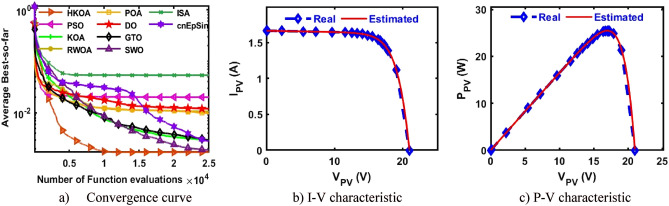
Comparison among algorithms under STM module based on SDM.

Table 10 displays the findings of used performance metrics after each algorithm was run 30 times on the SDM-based STM. This table demonstrates that HKOA is better than all the competing optimizers. We use the WRS test to further show that the results obtained by the HKOA are unique in comparison to the other algorithms. In Table 10, the p-value that the WRS test calculated when comparing HKOA to its competitors. This value shows that HKOA is significantly different from the other methods statistically. Additionally, the convergence curve is shown in Fig. 11a. This figure demonstrates that HKOA was able to attain the lowest RMSE after fewer than 10,000 function evaluations. Conversely, after completing the optimization process, some rival algorithms, including SWO, might be able to roughly match the least RMSE that HKOA was able to acquire. Therefore, HKOA is thought to be the optimum alternative for estimating the unknown parameters of the SDM-based STM module. I–V and P–V curves are shown in Fig. 11b,c using the best parameters found by HKOA, illustrating the similarity between the estimated and observed current.

**Table 10:** Comparison among algorithms using STM under SDM.

	Best-obtained parameters					RMSE				*P* value
	${I}_{ph}(A)$	${I}_{sd}(\mu A)$	${R}_{s}(\Omega )$	${R}_{sh}(\Omega )$	$n$	$Best$	*Worst*	*Avg*	*SD*	
HKOA	**1.6639**	**1.741E-06**	**0.0043**	**15.9315**	**1.5205**	**1.72192E-03**	**1.72192E-03**	**1.72192E-03**	**1.343E-17**	
PSO	**1.6639**	**1.741E-06**	**0.0043**	**15.9315**	**1.5205**	**1.72192E-03**	7.94295E-02	2.00613E-02	3.333E-02	**6.72E-04**
KOA	1.6627	2.644E-06	0.0028	18.2166	1.5678	1.99127E-03	3.78867E-03	2.98202E-03	4.465E-04	**3.02E-11**
RWOA	1.6625	3.101E-06	0.0023	19.5064	1.5867	2.19221E-03	7.94295E-02	1.05987E-02	2.335E-02	**3.01E-11**
POA	1.6593	5.932E-06	0.0000	31.2816	1.6682	3.49928E-03	1.34661E-02	9.68315E-03	4.174E-03	**3.02E-11**
DO	1.6644	1.316E-06	0.0052	14.8391	1.4902	1.83915E-03	7.94295E-02	1.20001E-02	1.329E-02	**3.02E-11**
GTO	**1.6639**	**1.741E-06**	**0.0043**	**15.9311**	**1.5205**	**1.72192E-03**	8.32547E-03	2.96518E-03	1.988E-03	**3.02E-11**
SWO	**1.6639**	**1.741E-06**	**0.0043**	**15.9315**	**1.5205**	**1.72192E-03**	3.16298E-03	1.88775E-03	3.513E-04	**1.89E-04**
ISA	1.6638	1.984E-06	0.0038	16.4010	1.5349	1.75164E-03	7.94295E-02	5.37547E-02	3.693E-02	**2.94E-11**
cnEpSin	**1.6639**	**1.741E-06**	**0.0043**	**15.9315**	**1.5205**	**1.72192E-03**	7.47877E-03	2.82568E-03	1.600E-03	**1.43E-05**

Bold font represents the best outcomes.

B. Double-Diode model

**Figure 12:**
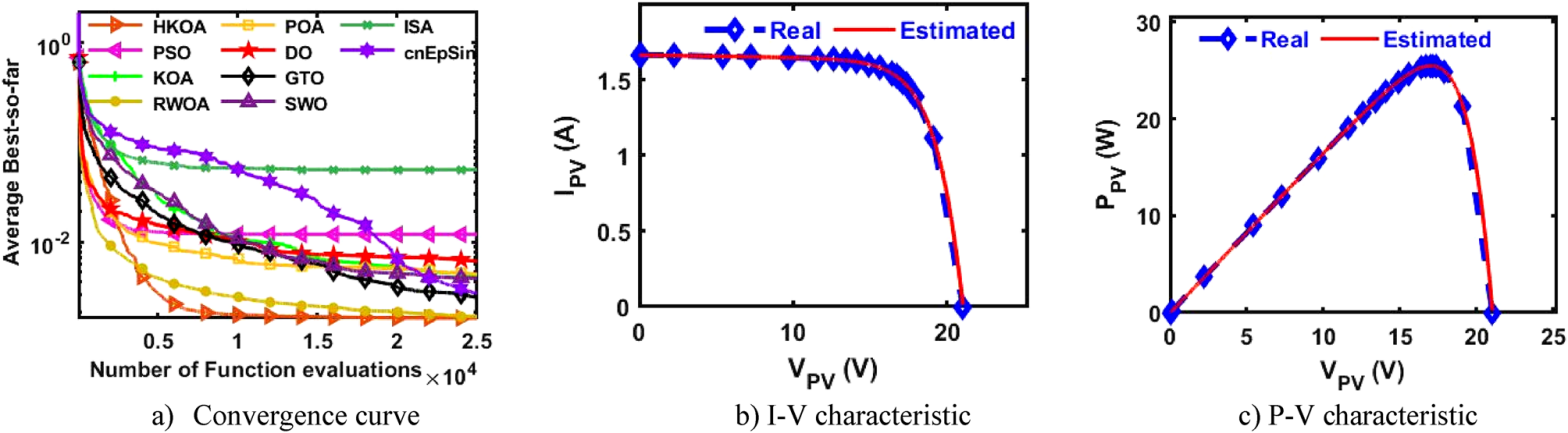
Comparison among algorithms under STM module based on DDM.

Table 11 summarizes the results of 30 times of each algorithm on the DDM-based STM in terms of a variety of performance measures. Based on these results, it appears that HKOA is the most effective algorithm, with RWOA coming in second and ISA coming in last. There is a statistically significant difference between HKOA and its competitors' outcomes, as illustrated by the p-value that is also reported in this table. Figure 12a depicts the convergence curve of each algorithm, further illustrating the HKOA's superiority. Broadly speaking, this figure shows that, after around 5,000 function evaluations, HKOA was able to achieve the lowest RMSE. On the other hand, after approximately 22,000 function evaluations, RWOA can roughly equal the lowest RMSE that HKOA was able to obtain. Consequently, HKOA is the best option for predicting the DDM-based STM module's unknown parameters. Figure 12b,c display the similarity between the measured data and the data estimated by HKOA.

**Table 11:** Comparison among algorithms using STM under DDM.

	Best-obtained parameters							RMSE				*P* value
	${I}_{ph}(A)$	${I}_{sd1}(A)$	${I}_{sd2}(A)$	${R}_{s}(\Omega )$	${R}_{sh}(\Omega )$	${{\text{n}}}_{1}$	${{\text{n}}}_{2}$	Best	Worst	Avg	SD	
HKOA	**1.664**	**4.31E-09**	**4.30E-06**	**0.007**	**17.407**	**1.105**	**1.703**	**1.676E-03**	**2.795E-03**	**1.735E-03**	**2.004E-04**	
PSO	1.664	1.00E-09	4.28E-06	0.008	17.696	1.028	1.699	1.678E-03	7.484E-02	1.199E-02	2.510E-02	**2.39E-04**
KOA	1.663	8.81E-07	4.07E-06	0.003	18.851	1.478	1.814	2.062E-03	3.014E-02	4.651E-03	5.421E-03	**7.39E-11**
RWOA	**1.664**	**1.00E-09**	**3.76E-06**	**0.008**	**17.245**	**1.031**	**1.675**	1.677E-03	3.238E-03	1.806E-03	3.039E-04	6.95E-01
POA	1.666	1.04E-09	1.68E-06	0.004	14.039	1.297	1.517	1.978E-03	7.603E-03	4.759E-03	1.622E-03	**4.98E-11**
DO	1.665	1.09E-09	1.64E-06	0.006	14.937	1.091	1.531	1.799E-03	1.304E-02	6.516E-03	2.788E-03	**4.08E-11**
GTO	1.664	1.37E-06	1.59E-06	0.004	16.120	1.498	1.974	1.715E-03	1.061E-02	2.810E-03	1.576E-03	**2.37E-10**
SWO	1.664	2.87E-07	4.92E-06	0.005	16.814	1.372	1.835	1.692E-03	7.484E-02	4.371E-03	1.333E-02	**5.61E-05**
ISA	1.664	4.61E-07	2.54E-06	0.005	16.290	1.419	1.720	1.705E-03	7.484E-02	5.300E-02	3.393E-02	**1.43E-10**
cnEpSin	**1.664**	**2.20E-09**	**3.68E-06**	**0.007**	**17.188**	**1.074**	**1.670**	**1.676E-03**	5.869E-03	3.093E-03	1.206E-03	**7.69E-08**

Bold font represents the best outcomes.

C. Triple-Diode model

**Figure 13:**
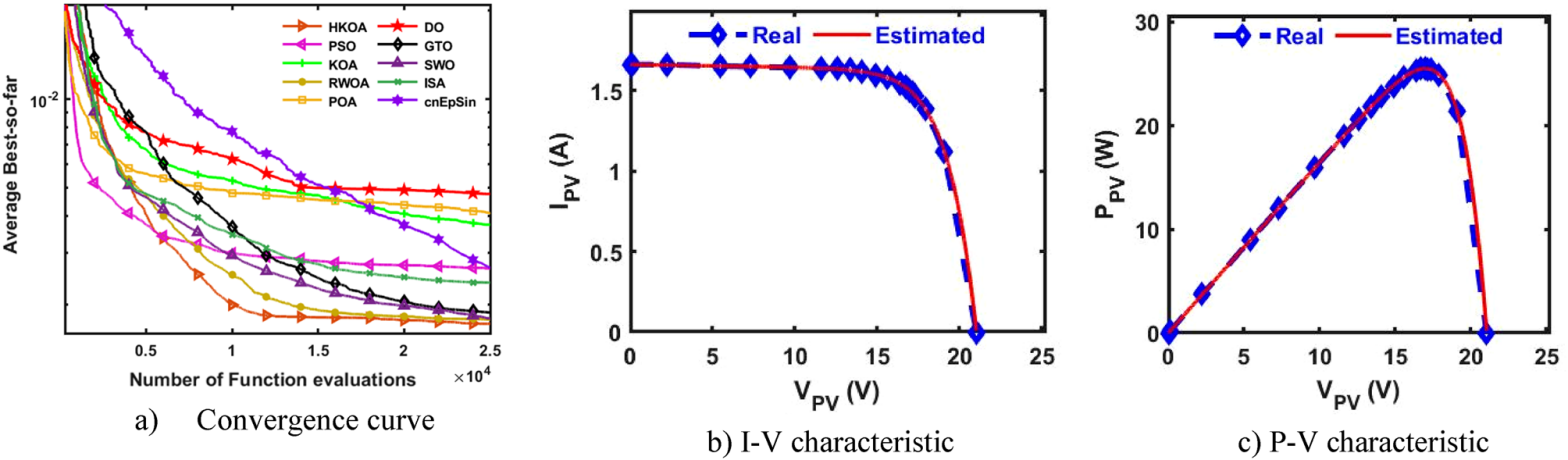
Comparison among algorithms under STM module based on TDM.

In this section, HKOA estimates nine unidentified TDM parameters based on the STM module to reveal its performance. Table 12 shows algorithm outcomes in this circumstance. HKOA performs best, followed by SWO, while DO is the worst. The p-value between HKOA and the rival algorithms shows that its results are distinct. Figure 13a shows each algorithm's convergence curves to show HKOA's supremacy. This figure, in general, indicates that HKOA was able to obtain the lowest RMSE following about 12,000 function evaluations. Conversely, following about 17,000 function evaluations, RWOA can roughly match the lowest RMSE that HKOA managed to achieve. For estimating the unknown characteristics of the TDM-based STM module, HKOA is therefore the most effective algorithm. Figure 13b,c illustrate that the HKOA-estimated I-V and P–V curves match the measured curves.

**Table 12:** Comparison among algorithms using STM under TDM.

	Best-obtained parameters									RMSE				*p *value
	${I}_{ph}(A)$	${I}_{sd1}(A)$	${I}_{sd2}(A)$	${I}_{sd3}(A)$	${R}_{s}(\Omega )$	${R}_{sh}(\Omega )$	${{\text{n}}}_{1}$	${{\text{n}}}_{2}$	${{\text{n}}}_{3}$	$Best$	Worst	Avg	SD	
HKOA	**1.663**	**2.4E-06**	**2.5E-08**	**3.9E-06**	**0.007**	**18.51**	**1.74**	**1.20**	**1.84**	**1.702E-03**	**1.75E-03**	**1.73E-03**	**1.5E-05**	
PSO	1.663	2.6E-08	7.4E-06	4.3E-07	0.006	18.17	1.24	1.95	1.46	1.708E-03	5.05E-03	2.66E-03	1.2E-03	**7.E-07**
KOA	1.662	1.4E-06	1.8E-07	3.8E-06	0.004	19.01	1.50	1.90	1.98	2.022E-03	4.70E-03	3.74E-03	6.7E-04	**3.E-11**
RWOA	1.663	1.0E-09	4.9E-07	5.6E-06	0.008	18.78	1.03	1.50	1.84	1.706E-03	1.92E-03	1.79E-03	8.2E-05	**4.E-03**
POA	1.663	6.1E-07	5.8E-08	2.4E-06	0.004	16.31	1.45	1.56	1.73	2.188E-03	5.29E-03	4.09E-03	1.1E-03	**3.E-11**
DO	1.663	1.3E-08	2.0E-06	2.8E-09	0.004	17.11	1.58	1.54	1.48	1.769E-03	1.22E-02	4.76E-03	2.2E-03	**3.E-11**
GTO	1.663	1.0E-09	4.7E-06	1.5E-07	0.007	18.33	1.04	1.76	1.41	1.705E-03	2.89E-03	1.88E-03	2.8E-04	**5.E-06**
SWO	1.663	2.5E-07	9.8E-06	3.4E-08	0.006	18.34	1.35	2.00	1.56	1.707E-03	3.32E-03	1.80E-03	2.9E-04	**5.E-02**
ISA	1.663	1.0E-09	8.2E-07	5.2E-06	0.005	17.47	1.99	1.45	2.00	1.726E-03	4.71E-03	2.38E-03	6.4E-04	**2.E-10**
cnEpSin	1.663	7.9E-06	6.3E-08	4.4E-07	0.005	17.94	2.00	2.00	1.40	1.713E-03	3.91E-03	2.67E-03	7.0E-04	**8.E-08**

Bold font represents the best outcomes.

### Kyocera KC200GT module

**Figure 14:**
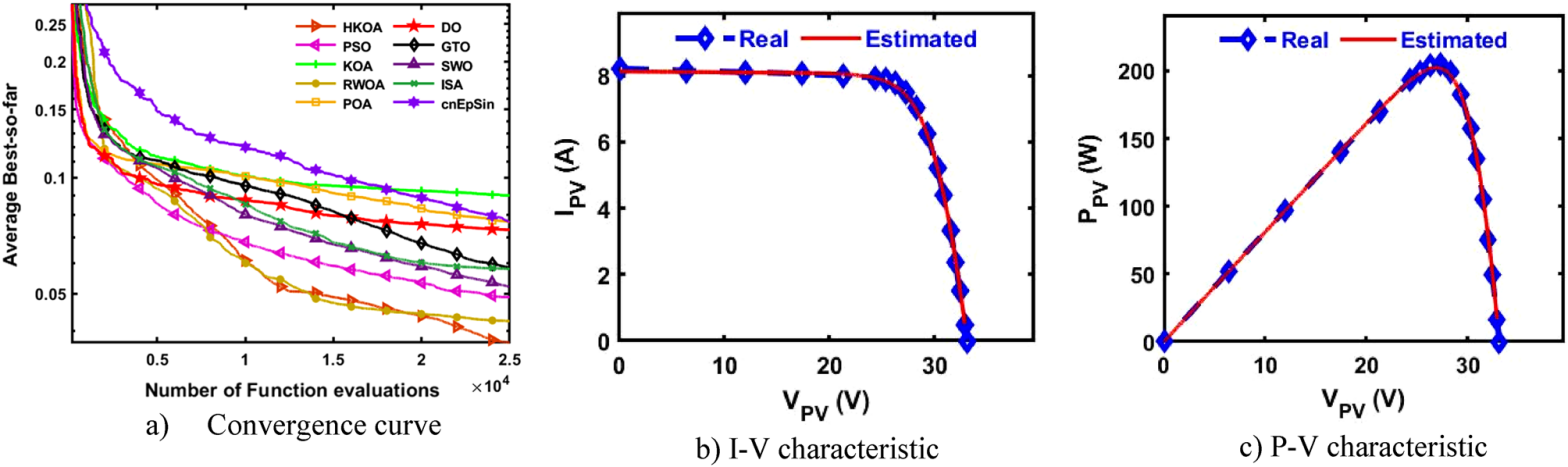
Comparison among algorithms under KKC based on TDM.

Due to the high accuracy of TDM over both DDM and SDM, in this section and the next two sections, we will further focus on observing the performance of HKOA for estimating its nine known parameters under three different PV modules. After 30 independent trials for each algorithm on the TDM-based KKC, a number of performance indicators are estimated and reported in Table 13. This table illustrates that HKOA ranks first, RWOA is the second best, and POA is the worst algorithm. This table also provides the p-value that compares HKOA's findings to those of each algorithm; this value demonstrates that HKOA's results are unique from those of all other algorithms. Figure 14a illustrates the convergence curves for each algorithm to further affirm HKOA's supremacy; this figure illustrates HKOA's superiority. In addition, Fig. 14b,c are presented to demonstrate that the I-V and P–V curves estimated by HKOA and the measured curves are consistent with one another.

**Table 13:** Comparison among algorithms using KKC under TDM.

	Best-obtained parameters									RMSE				
	${I}_{ph}(A)$	${I}_{sd1}(A)$	${I}_{sd2}(A)$	${I}_{sd3}(A)$	${R}_{s}(\Omega )$	${R}_{sh}(\Omega )$	${{\text{n}}}_{1}$	${{\text{n}}}_{2}$	${{\text{n}}}_{3}$	$Best$	Worst	Avg	SD	p-value
HKOA	**8.201**	**1.0E-09**	**1.0E-09**	**1.0E-09**	**0.005**	**2.64**	**1.05**	**2.00**	**2.00**	**2.821E-02**	**6.84E-02**	**3.73E-02**	**1.2E-02**	
PSO	8.161	1.0E-09	1.2E-07	2.2E-08	0.005	3.66	1.05	1.98	1.53	3.085E-02	8.40E-02	4.90E-02	1.2E-02	**8.E-06**
KOA	8.143	4.6E-07	2.9E-07	4.8E-07	0.003	451.24	1.96	1.39	1.98	7.051E-02	1.08E-01	8.99E-02	7.2E-03	**3.E-11**
RWOA	**8.201**	**1.0E-09**	**1.0E-09**	**1.0E-09**	**0.005**	**2.64**	**1.05**	**2.00**	**2.00**	**2.821E-02**	6.84E-02	4.25E-02	1.1E-02	**6.E-03**
POA	8.132	1.0E-09	4.5E-06	2.4E-07	0.004	167.88	1.06	1.93	1.58	5.079E-02	1.01E-01	7.67E-02	1.4E-02	**2.E-10**
DO	8.104	2.3E-09	4.9E-09	1.9E-06	0.004	356.71	1.09	1.80	1.86	4.410E-02	1.04E-01	7.32E-02	1.9E-02	**9.E-10**
GTO	8.193	1.0E-09	1.0E-08	5.4E-08	0.005	2.86	1.05	2.00	2.00	2.848E-02	7.85E-02	5.85E-02	1.2E-02	**7.E-08**
SWO	8.192	1.8E-09	7.4E-08	2.3E-08	0.004	2.94	1.07	1.98	1.97	2.979E-02	6.84E-02	5.21E-02	1.3E-02	**2.E-06**
ISA	8.210	1.0E-09	1.0E-09	1.0E-09	0.005	2.51	1.05	1.61	1.40	2.842E-02	7.60E-02	5.80E-02	1.2E-02	**1.E-07**
cnEpSin	8.185	4.7E-09	1.2E-08	8.9E-08	0.004	3.96	1.13	1.82	1.43	4.307E-02	1.04E-01	7.67E-02	1.3E-02	**2.E-10**

Bold font represents the best outcomes.

### Ultra 85-P module

**Figure 15:**
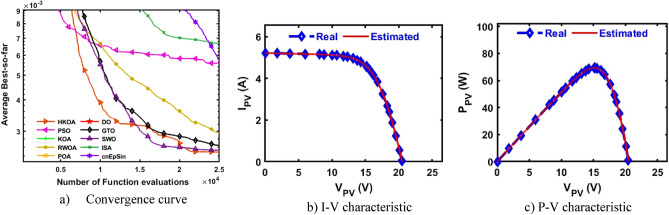
Comparison among algorithms under Ultra 85-P based on TDM.

To further study the HKOA’s performance over TDM, in this section, an additional PV module called Ultra 85-P is utilized. The findings obtained by various algorithms over this module are displayed in Table 14. The algorithm with the best performance, according to this table, is HKOA, followed by SWO, and POA is the worst. This table also contains the p-value between HKOA and each rival optimizer; this value indicates that HKOA's results are distinct from those of all other algorithms. Figure 15a depicts the convergence curves for each algorithm to further illustrate HKOA's superiority; this figure shows HKOA's superiority. In addition, Fig. 15b,c illustrate the similarity between the I–V and P–V curves estimated by HKOA and the measured curves.

**Table 14:** Comparison among algorithms using Ultra under TDM.

	Best-obtained parameters									RMSE				p-value
	${I}_{ph}(A)$	${I}_{sd1}(A)$	${I}_{sd2}(A)$	${I}_{sd3}(A)$	${R}_{s}(\Omega )$	${R}_{sh}(\Omega )$	${{\text{n}}}_{1}$	${{\text{n}}}_{2}$	${{\text{n}}}_{3}$	$Best$	Worst	Avg	SD	
HKOA	**5.226**	**1.7E-06**	**1.0E-05**	**1.0E-05**	**0.011**	**3.99**	**1.43**	**1.72**	**1.92**	**2.427E-03**	**2.55E-03**	**2.48E-03**	**3.3E-05**	
PSO	5.226	1.0E-05	7.6E-06	4.2E-06	0.011	3.95	1.85	1.96	1.49	2.446E-03	1.73E-02	5.57E-03	4.7E-03	**1.E-05**
KOA	5.215	6.4E-06	7.8E-08	8.1E-06	0.011	4.73	1.72	1.60	1.56	5.152E-03	1.51E-02	9.13E-03	2.5E-03	**3.E-11**
RWOA	5.227	1.8E-06	1.0E-05	1.0E-05	0.011	3.91	1.42	1.79	1.84	2.459E-03	5.92E-03	2.96E-03	7.4E-04	**4.E-07**
POA	5.219	6.3E-06	2.4E-06	1.8E-06	0.011	4.23	1.59	1.82	1.46	5.163E-03	2.17E-02	1.43E-02	4.6E-03	**3.E-11**
DO	5.220	8.0E-06	8.8E-06	3.7E-08	0.011	4.43	1.55	1.82	1.74	3.197E-03	3.25E-02	1.16E-02	8.4E-03	**3.E-11**
GTO	5.226	1.4E-06	6.9E-06	1.0E-05	0.011	3.91	1.44	1.61	1.93	2.463E-03	3.78E-03	2.64E-03	2.9E-04	**2.E-05**
SWO	5.226	7.1E-06	9.9E-06	1.3E-06	0.011	3.94	1.69	1.79	1.41	2.438E-03	3.45E-03	2.52E-03	1.8E-04	**6.E-01**
ISA	5.226	4.3E-06	6.3E-07	6.5E-06	0.011	3.86	1.53	1.76	1.62	2.544E-03	1.79E-02	6.69E-03	4.2E-03	**3.E-11**
cnEpSin	5.229	1.1E-06	9.9E-08	9.2E-06	0.011	3.64	1.94	1.80	1.56	2.684E-03	9.06E-03	5.69E-03	2.0E-03	**3.E-11**

Bold font represents the best outcomes.

### STP module

**Figure 16:**
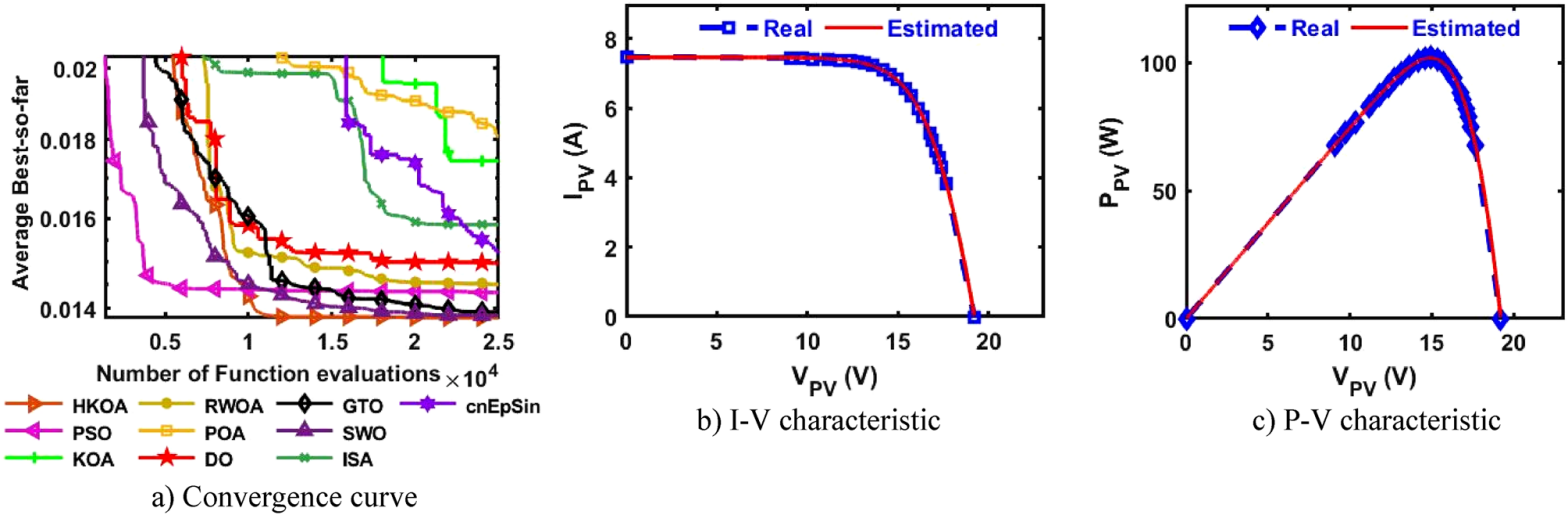
Comparison among algorithms under STP based on TDM.

Finally, in this section, the STP module is utilized to further study the HKOA's performance over TDM. Table 15 displays the results acquired by various algorithms on this module. According to this table, the algorithm with the greatest performance is HKOA, followed by SWO, while DO is classified as the worst algorithm. This table also includes the p-value between HKOA and each algorithm compared; this value indicates that HKOA's results are distinct from all other algorithms. Figure 16a illustrates the convergence curves for each algorithm to further demonstrate HKOA's superiority; this figure demonstrates HKOA's superiority. Moreover, Fig. 16b,c depict the similarity between the I-V and P–V curves estimated by HKOA and the measured curves.

**Table 15:** Comparison among algorithms using STP under TDM.

	Best-obtained parameters									RMSE				p-value
	${I}_{ph}(A)$	${I}_{sd1}(A)$	${I}_{sd2}(A)$	${I}_{sd3}(A)$	${R}_{s}(\Omega )$	${R}_{sh}(\Omega )$	${{\text{n}}}_{1}$	${{\text{n}}}_{2}$	${{\text{n}}}_{3}$	$Best$	Worst	Avg	SD	
HKOA	**7.476**	**1.0E-09**	**1.9E-06**	**1.0E-09**	**0.005**	**15.14**	**1.24**	**1.24**	**2.00**	**1.380E-02**	**1.39E-02**	**1.38E-02**	**2.3E-05**	
PSO	7.476	2.2E-08	1.9E-06	5.6E-08	0.005	15.22	1.15	1.25	1.92	1.381E-02	1.55E-02	1.42E-02	4.0E-04	**1.E-10**
KOA	7.463	1.4E-06	5.3E-06	2.5E-06	0.005	55.80	1.22	1.94	1.59	1.426E-02	1.86E-02	1.54E-02	1.1E-03	**3.E-11**
RWOA	7.480	1.9E-06	1.1E-09	1.0E-09	0.005	12.84	1.24	2.00	1.40	1.382E-02	1.55E-02	1.45E-02	5.8E-04	**9.E-11**
POA	7.459	5.1E-09	2.2E-06	1.2E-06	0.005	475.82	1.46	1.25	1.94	1.426E-02	1.91E-02	1.55E-02	1.1E-03	**3.E-11**
DO	7.468	6.1E-08	9.8E-07	6.9E-06	0.005	41.78	1.99	1.20	1.61	1.430E-02	3.00E-02	1.75E-02	3.2E-03	**3.E-11**
GTO	**7.476**	**9.9E-09**	**1.9E-06**	**1.3E-09**	**0.005**	**15.31**	**1.92**	**1.24**	**1.96**	**1.380E-02**	1.49E-02	1.40E-02	2.9E-04	**1.E-09**
SWO	**7.476**	**1.9E-06**	**4.6E-08**	**1.0E-09**	**0.005**	**15.14**	**1.24**	**1.24**	**2.00**	**1.380E-02**	1.50E-02	1.39E-02	2.2E-04	**1.E-04**
ISA	7.476	1.9E-06	1.9E-08	2.3E-07	0.005	15.44	1.24	1.99	1.64	1.381E-02	2.71E-02	1.48E-02	2.5E-03	**9.E-11**
cnEpSin	7.467	4.5E-09	2.0E-06	2.1E-06	0.005	34.04	1.26	1.25	1.94	1.422E-02	1.78E-02	1.49E-02	7.1E-04	**3.E-11**

Bold font represents the best outcomes.

### Computational cost

**Figure 17:**
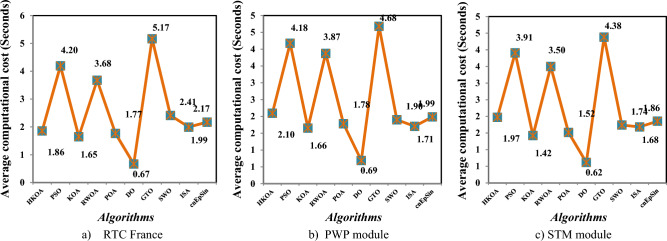
Comparison between HKOA and its competitors in terms of computational cost over TDM.

After clarifying the HKOA’s effectiveness in the former sections, it is time to show its efficiency under the computational cost consumed from the beginning of the optimization process to the end. Therefore, the average computational costs for each algorithm within 30 independent times on various PV modules based on TDM are computed and reported in Fig. 17. According to this figure, the computational cost for all algorithms is nearly competitive, except for DO, which consumes nearly a third of the computational cost of the others. Despite that, DO could not be considered a strong alternative for extracting the unknown parameters of various PV models because it has weak performance compared to HKOA. In general, since HKOA could achieve outstanding outcomes in a reasonable time, it is considered a strong alternative for tackling the parameter estimation problem of PV models.

### HKOA’s sensitivity analysis

**Figure 18:**
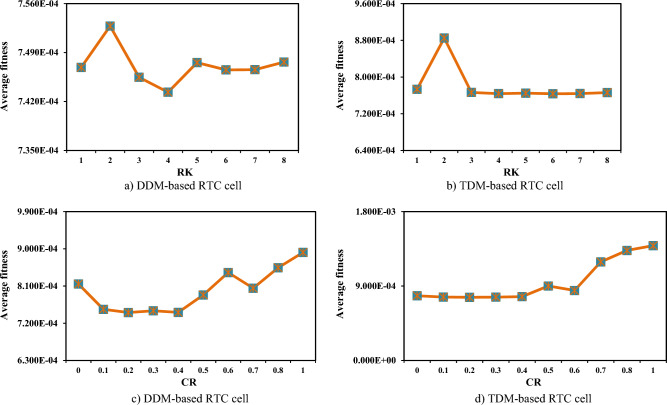
HKOA’s parameter tuning.

Accurate determination of the best values for the newly proposed controlling parameters, namely RK and CR, is an essential step to maximize the performance of HKOA when applied to dealing with parameter estimation of the PV models. Therefore, extensive experiments were performed on a variety of PV models under a wide range of values, and the results of those experiments are summarized and presented in Fig. 18. Observing this figure illustrates that the HKOA performance is significantly enhanced when RK and CR are 4 and 0.2, respectively. The classical KOA’s parameters are all set according to what is recommended in^46^. In brief, HKOA’s parameters are listed in Table 16.

**Table 16:** HKOA’s parameters.

Parameter	Value
*N*	25
*M*_*0*_	0.5
*λ*	10
*T*	3
*CR*	0.2
*RK*	4

## Conclusions and future works

This paper introduces a new technique for approximating the unidentified parameters of three PV models, namely TDM, DDM, and SDM. This technique is called a hybrid KOA and is based on integrating the recently proposed KOA with two effective mechanisms, namely the ranking-based update and exploitation improvement mechanisms, to enhance its exploration and exploitation capabilities for accurately solving this complex optimization problem. The first mechanism is used to promote the KOA’s exploration operator to diminish getting stuck in local optima, while the second mechanism promotes the exploitation operator to fulfill a better solution in smaller function evaluations. Both KOA and HKOA are verified using the RTC France solar cell and five PV modules, and their effectiveness is determined by comparing them to eight rival algorithms. Experimental findings indicate that HKOA is the most effective method for parameter estimation of TDM, DDM, and SDM since it could produce significantly different and superior results for various tackled PV modules. Although the proposed HKOA could achieve outstanding outcomes for this problem, it still suffers from some shortcomings, such as the parameter tuning problem and a slightly high computational cost, which might affect its performance when applied to other real applications. Therefore, in the future, we will design new search mechanisms based on chaotic maps or opposition-based theory to improve the performance of KOA in terms of reducing the additional control parameters and minimizing high computational requirements. In addition, our future work will investigate the performance of HKOA for several other optimization problems like estimating the unknown parameters of fuel cells, image denoising, image segmentation, image registration, image enhancement, image fusion, and feature selection.

## Data Availability

The datasets used and/or analysed during the current study available from the corresponding author on reasonable request.

## List of symbols

$${I}_{ph}$$: Photocurrent
$${I}_{D}$$: Diode current
$$k$$: Boltzmann constant
$$V$$: Output voltage
$${R}_{sh}$$: Parallel resistance
$$N$$: Population size
$d$: Number of dimensions
${T}_{i}$: Orbital period of *i*th planet
${V}_{i}$: Velocity of $ith$ planet
${I}_{sd}, {I}_{sd1}, {I}_{sd2}, {I}_{sd3}$: Reverse saturation current
$${R}_{s}$$: Series resistance
$$q$$: Electron charge
$T$: Temperature in kelvin
$$n, {n}_{1}, {n}_{2}, {n}_{3}$$: Ideality factor
$$N$$: Population size
$$e$$: Orbital eccentricity
$${\text{F}}$$: Gravitational force
${T}_{{\text{max}}}$: The maximum function evaluation

## Abbreviations

PV: Photovoltaic
KOA: Kepler optimization algorithm
DDM: Double diode model
SDM: Single diode model
TDM: Triple diode model
RMSE: Root mean squared error
STP6: STP6-120/36 module
STM: STM6-40/36 module
NR: Newton–Raphson
MPPT: Maximum power point tracking
MPA: Marine predators algorithm (MPA)
OF: Objective function
CR: Convergence rate
STC: Standard conditions
Ultra: Ultra 85-P module
PWP: Photowatt-PWP201 module
KKC: Cc module
RUM: Ranking-based updating method
WRS: Wilcoxon rank-sum
